# Design, Synthesis, and Evaluation of a New Chemotype Fluorescent Ligand for the P2Y_2_ Receptor

**DOI:** 10.1021/acsmedchemlett.4c00211

**Published:** 2024-06-12

**Authors:** Rebecca Knight, Laura E. Kilpatrick, Stephen J. Hill, Michael J. Stocks

**Affiliations:** † Division of Biomolecular Sciences and Medicinal Chemistry, School of Pharmacy, University of Nottingham, Nottingham NG7 2RD, U.K.; ‡ Centre of Membrane Proteins and Receptors (COMPARE), Universities of Birmingham and Nottingham, The Midlands NG7 2UH, U.K.; § Division of Physiology, Pharmacology and Neuroscience, School of Life Sciences, University of Nottingham, Nottingham NG7 2UH, U.K.

**Keywords:** Antagonists, Fluorescence, Ligands, Receptors

## Abstract

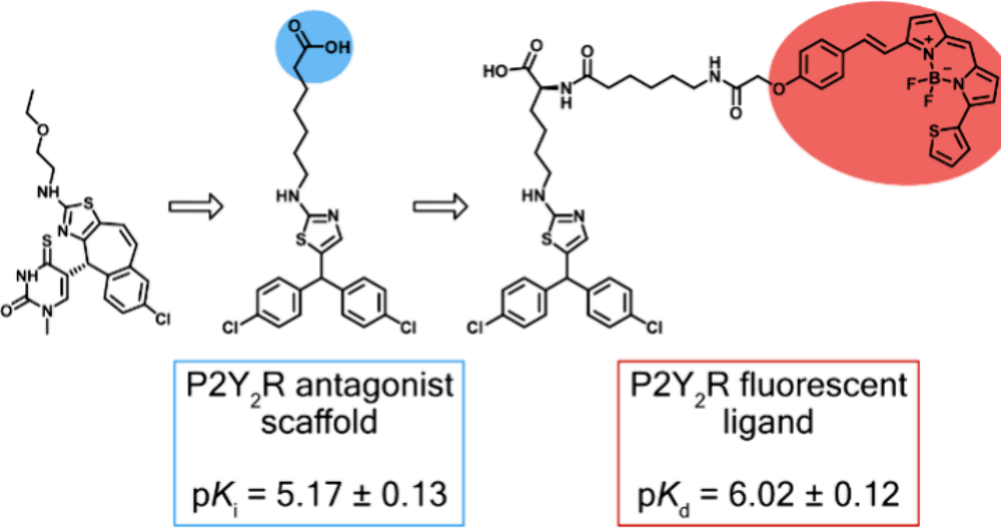

The P2Y_2_ receptor (P2Y_2_R) is a target for diseases including cancer, idiopathic pulmonary fibrosis, and atherosclerosis. However, there are insufficient P2Y_2_R antagonists available for validating P2Y_2_R function and future drug development. Evaluation of how (*R*)-5-(7-chloro-2-((2-ethoxyethyl)amino)-4*H*-benzo[5,6]cyclohepta[1,2-*d*]thiazol-4-yl)-1-methyl-4-thioxo-3,4-dihydropyrimidin-2(1*H*)-one, a previously published thiazole-based analogue of AR-C118925, binds in a P2Y_2_R homology model was used to design new P2Y_2_R antagonist scaffolds. One P2Y_2_R antagonist scaffold retained millimolar affinity for the P2Y_2_R and upon further functionalization with terminal carboxylic acid groups affinity was improved over 100-fold. This functionalized P2Y_2_R antagonist scaffold was employed to develop new chemotype P2Y_2_R fluorescent ligands, that were attainable in a convergent five-step synthesis. One of these fluorescent ligands demonstrated micromolar affinity (p*K*_d_ = 6.02 ± 0.12, *n* = 5) for the P2Y_2_R in isolated cell membranes and distinct pharmacology from an existing P2Y_2_R fluorescent antagonist, suggesting it may occupy a different binding site on the P2Y_2_R.

The P2Y receptors (P2YRs) are a family of eight G protein-coupled receptors (GPCRs), found in almost all cell and tissue types, which mediate the signaling of nucleotides.^1^ The P2Y_2_ receptor (P2Y_2_R) is grouped with the “P2Y_1_-like” receptors according to its sequence homology and primary coupling to G_q_.^1^ However, the P2Y_2_R is uniquely activated by both adenosine-5′-triphosphate (ATP) and uridine-5′-triphosphate (UTP) at equivalent concentrations.^1^ P2Y_2_R signaling through G_q_ stimulates phospholipase C activity and the induction of the secondary messengers inositol-1,4,5-trisphosphate and diacylglycerol, which coordinate the release of Ca^2+^ ions from intracellular stores and activate protein kinase C, respectively.^2^ The P2Y_2_R is also coupled to the G_o_ and G_12_ proteins, which activate Rac and Rho GTPases, and can signal through Src kinase.^3−5^

Activation of the P2Y_2_R has been identified as contributing to several clinical conditions. The P2Y_2_R stimulated cytosolic phospholipase A_2_ and arachidonic acid release, which is subsequently metabolized to inflammatory molecules like prostaglandins, and are involved in diseases associated with chronic inflammation.^6−9^ In mouse models activation of the P2Y_2_R induced vascular inflammation and atherosclerosis, with increased uptake of low-density lipoprotein in vascular smooth muscle.^10−12^ While in mouse models of idiopathic pulmonary fibrosis P2Y_2_R deficiency reduced inflammation and fibrosis, preventing an ATP driven increase in macrophages and neutrophils, and migration of fibroblasts.^13^ In cancer, upon stimulation by ATP released from tumor cell-activated platelets, endothelial P2Y_2_R facilitated extravasation of tumor cells at metastatic sites by opening the endothelial barrier.^14^ In several mouse models including breast and lung cancer, the knockout and/or pharmacological inhibition of the P2Y_2_R decreased tumor growth and reduced metastasis.^14−18^ Despite being a promising therapeutic target, there is a lack of desirable P2Y_2_R antagonists available for drug development or further pharmacological evaluation of P2Y_2_R function and signaling.

AR-C118925 (**1**) is the most potent and selective antagonist for the P2Y_2_R, developed from UTP, with a midnanomolar IC_50_ and 50- to 500-fold selectivity over other P2YR subtypes.^20,23^ Despite being a helpful pharmacological tool, AR-C118925 had limited bioavailability when administered orally in preclinical studies, reflecting its poor physiochemical properties.^20^ In a P2Y_2_R homology model, docking studies suggested AR-C118925 bound in the orthosteric site and that occupation of a lipophilic binding pocket by the tricyclic 2,8-dimethyl-5*H*-dibenzo[*a,d*][7]annulene group conferred high receptor affinity and antagonist activity in calcium mobilization assays.^21,24^ A series of AR-C118925 analogues have been detailed which replaced this moiety with 7-chloro-4*H*-benzo[5,6]cyclohepta[1,2-*d*]thiazole to reduce lipophilicity.^19^ The inclusion of linear, nonsterically demanding substituents on the thiazole ring increased affinity (for example compound **2**) and provided a linking site for the attachment of fluorophores, generating the first fluorescent antagonist for the P2Y_2_R with micromolar affinity (**3**) (Figure 1).^19^ In bioluminescence resonance energy transfer (BRET)-based assays, fluorescent antagonist **3** was used with P2Y_2_R tagged on its *N*-terminus with NanoLuciferase (NLuc) to determine the affinities of several unlabeled P2Y_2_R antagonists in NanoBRET competition binding experiments.^19^ Fluorescent antagonist **3** provides an indispensable tool to identify new P2Y_2_R antagonists with the distance and orientation proximity required for NanoBRET (10 nm) allowing determination of binding for low affinity compounds.^19,25^

**Figure 1:**
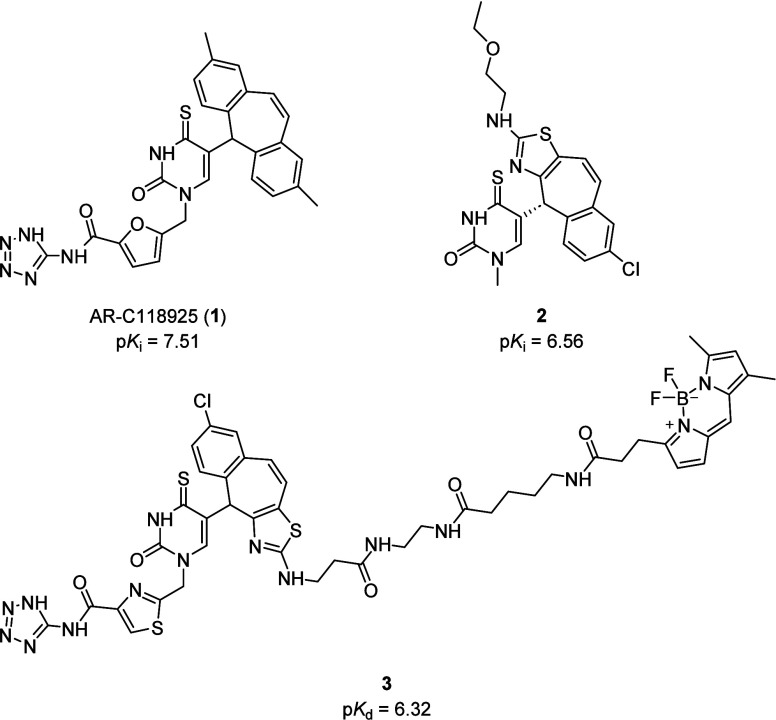
Illustrative structure for the thiazole-based series of P2Y_2_ receptor antagonists (**2**), based on AR-C118925 (**1**), and the first fluorescent antagonist for the P2Y_2_ receptor (**3**).^19,20^

In the present study, the binding pose of **2**, a thiazole-based analogue of **1**, was evaluated in a P2Y_2_R homology model and used to design a new series of P2Y_2_R antagonist scaffolds.^19,21^ Herein, we report the design, synthesis, and pharmacological evaluation of these P2Y_2_R antagonist scaffolds and their subsequent development into new chemotype fluorescent ligands that bind to the P2Y_2_R in membranes.

The synthetic strategy began with investigation of how **2**, the highest affinity compound in the thiazole-based series of P2Y_2_R antagonists, might bind in a P2Y_2_R homology model.^19,21^ Induced-fit docking simulations for **2** demonstrated a different binding pose to **1**, with the thiouracil ring partially occupying the lipophilic binding pocket (Leu89, Phe113, Tyr114, Val168, Phe171, Val172, Phe195, and Phe261) and the thiazole substituent extended upward toward a basic amino acid triad (His184, Arg272, and Arg24) (Figure 2).^21,22^ Interestingly, the tricyclic 7-chloro-4*H*-benzo[5,6]cyclohepta[1,2-*d*]thiazole group is not anchored in the lipophilic binding pocket, despite these interactions being predicted as crucial for the binding of **1** (p*K*_i_ = 7.38 ± 0.04).^19,21^ The observed change in binding pose could result from steric constraints imposed by the linear thiazole substituent; more sterically demanding substituents were inactive at the P2Y_2_R.^19^ Despite this different predicted binding pose, **2** retains good affinity for the P2Y_2_R (p*K*_i_ = 6.56 ± 0.16) which suggests replacement of the tricyclic ring system and thiouracil might be tolerated to generate a series of P2Y_2_R antagonists with improved physiochemical properties.^19^

**Figure 2:**
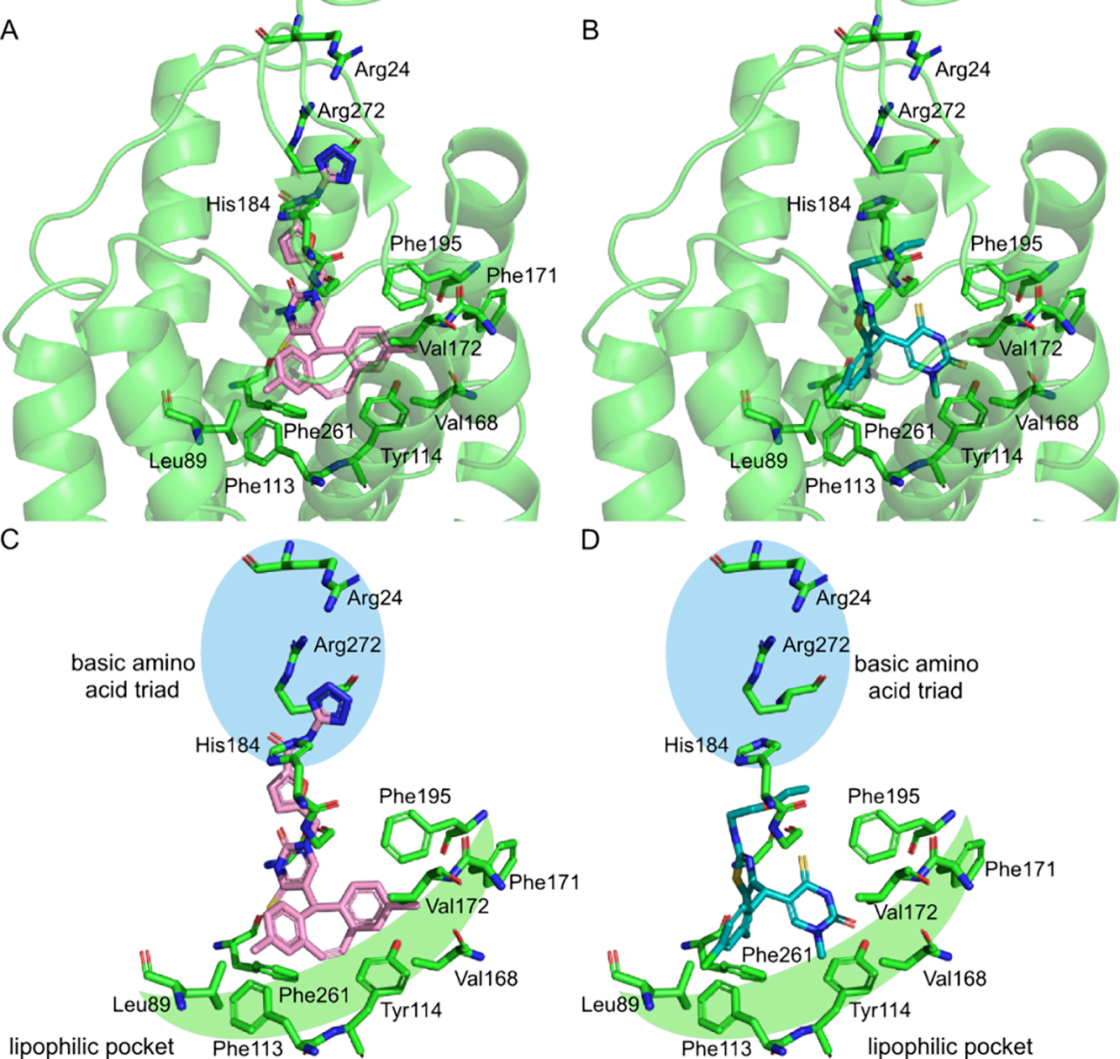
Putative binding pose of AR-C118925 (A + C) and thiazole-based analogue **2** (B + D) in a published homology model of the P2Y_2_ receptor with important residues in the binding pocket shown.^21^ The receptor is shown in cartoon representation, while amino acids (green), AR-C118925 (pink), and **2** (teal) are displayed as stick models. Oxygen atoms are shown in red, nitrogen in blue, chlorine in green, and sulfur in yellow. Docking experiments were performed using OEDocking Hybrid Docking.^22^ Homology model kindly supplied by Dr Müller.^21^

To test this hypothesis, a series of potential P2Y_2_R antagonists with sequential replacement of the tricyclic ring system and thiouracil were designed (Figure 3). Another predicted key interaction for the potency of **1**, is the formation of ionic salt bridges between the acylated tetrazole, which would be deprotonated at physiological pH, and a basic amino acid triad (His184, Arg272, and Arg24) (Figure 2).^21^ In the binding pose of **2** the linear thiazole substituent 2-ethoxyethylamine extends upward toward the basic amino acid triad, and presents an opportunity to recapitulate the interactions of **1** with these amino acids through the incorporation of acidic groups. Thus, with an aim to improve affinity, any active P2Y_2_R antagonist scaffolds would be functionalized with thiazole substituents containing terminal acidic groups (Figure 3).

**Figure 3:**
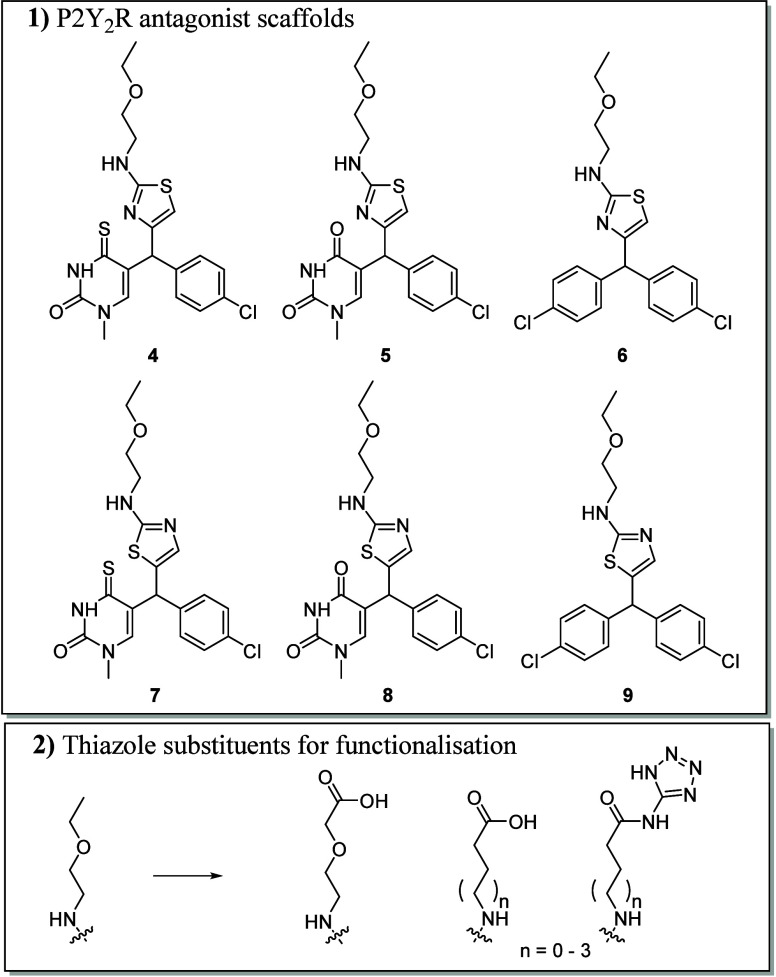
Structure of potential P2Y_2_R antagonists.

The synthesis pathway to reach compounds **4**-**8** is shown in Scheme 1. The ethyl 2-chlorothiazole-4-carboxylate (**10**) and ethyl 2-chlorothiazole-5-carboxylate (**17**) were hydrolyzed to afford the carboxylic acids **11** and **18**. Treatment of **11** and **18** with oxalyl chloride generated the acid chlorides, which were immediately converted to the Weinreb amides **12** and **19**. The Weinreb amides were then reacted with Grignard reagent, 4-chlorphenylmagnesium bromide, to provide the ketones **13** and **20** in a single addition. In the next step, 5-bromo-2,4-di-*ter*t-butoxypyrimidine was lithiated with *n*-butyllithium and then a 1,2-addition gave the tertiary alcohols **14** and **21**. Subsequent deprotection and reduction was achieved with triethylsilane and trifluoracetic acid to give **15** and **22**. Alkylation at the *N*-1 position with iodomethane in a one-pot process that first involved reversible silylation of the carbonyl groups on the uracil, using *N,O*-*bis*(trimethylsilyl)trifluoroacetamide, was carried out to give **16** and **23**. The thiazole chlorine atoms of **16** and **23** were displaced with 2-ethoxyethylamine upon heating in the microwave under basic conditions to give **5** and **8**. Finally, reaction with Lawesson’s reagent provided the 4-thiouracils **4** and **7**.

**Scheme 1:**
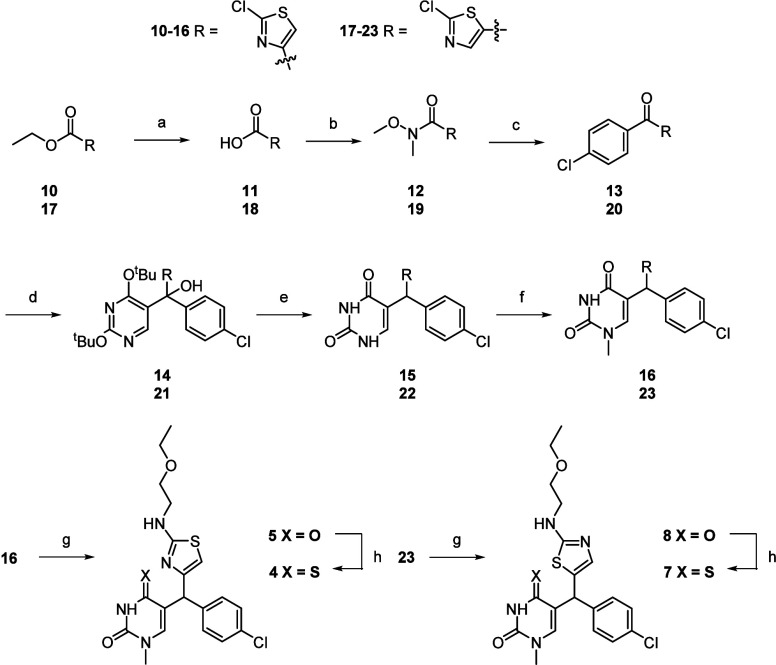
Synthesis of Compounds **4**–**23** Reagents and conditions: (a) NaOH, THF/H_2_O, rt, overnight (98%). (b) (i) Oxalyl chloride, catalytic DMF, THF, rt, 5 h (ii) DIPEA, *N*,*O*-dimethylhydroxylamine-HCl, DCM, rt, overnight (99% over two steps). (c) 4-Chlorophenylmagnesium bromide, THF, −78 °C to rt, overnight (40–62%). (d) (i) 5-Bromo-2,4-di-*ter*t-butoxypyrimidine, *n*-butyllithium, THF, −78 °C, 15 min; (ii) **14**, – 78 °C to rt, 1 h (17–55%). (e) TFA, triethylsilane, DCM, rt, 30 min (16–71%). (f) (i) *N,O*-Bis(trimethylsilyl)trifluoroacetamide, 1,2-dichloroethane, reflux, 18 h; (ii) iodomethane, 50 °C, 18 h (62%). (g) 2-Ethoxyethylamine, triethylamine, DMSO, 150 °C, 30 min (26–45%). (h) Lawesson’s reagent, 1,4-dioxane, reflux, 18 h (4–43%).

The synthesis pathway of compounds **6** and **9** is illustrated in Scheme 2. Compounds **10** and **17** were treated with excess Grignard reagent, 4-chlorophenylmagnesium bromide, to facilitate two step addition, to the ester and then ketone, formed through elimination, to give the tertiary alcohols **24** and **26**. Subsequent reduction with triethylsilane and trifluoracetic acid afforded **25** and **27**. The thiazole chlorine atoms of **25** and **27** were readily displaced with 2-ethoxyethylamine upon heating in the microwave under basic conditions to give **6** and **9**.

**Scheme 2:**
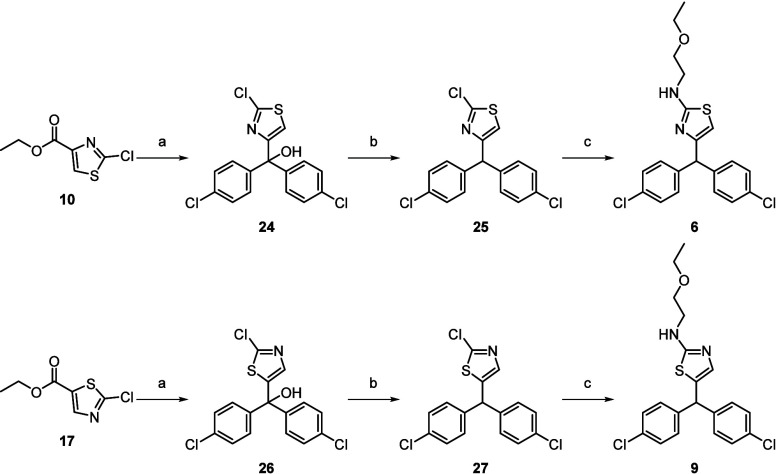
Synthesis of Compounds **6**–**27** Reagents and conditions: (a) 4-Chlorophenylmagnesium bromide, THF, −78 °C to rt, overnight (58–63%). (b) TFA, triethylsilane, DCM, rt, 30 min (82–84%). (c) 2-Ethoxyethylamine, triethylamine, DMSO, 150 °C, 30 min (53–57%).

These potential P2Y_2_R antagonist scaffolds were then evaluated using NanoBRET to assess their ability to displace fluorescent antagonist **3**, and consequently reduce the BRET ratio, at a single antagonist point concentration of 100 μM (Table 1). These experiments were performed in membrane preparations made from clonal 1321N1 cells stably expressing the NLuc tagged P2Y_2_R. Of the new P2Y_2_R antagonist scaffolds only compound **9** significantly reduced the BRET ratio (** *P* < 0.01 one-way ANOVA with Tukey’s multiple comparison test). Interestingly compound **6**, the regioisomer of **9**, demonstrated no reduction of the BRET ratio suggesting that the position of the thiazole ring is important for retaining binding. This could reflect either the formation of a key interaction with the thiazole group or a conformational preference for one regioisomer. However, **9** was not soluble at the higher concentrations required to complete a dose–response competition binding assay. We estimate that compound **9** had a p*K*i = ∼ 3 because the BRET ratio could be reduced by 44% at 1 mM (Supplementary Figure 1).

**Table 1:** Affinities for New P2Y_2_ Receptor Antagonists

Compound	p*K*_i_ ± (SEM)^a^/% BRET signal inhibition at 100 μM^b^	% calcium mobilization inhibition at 10 μM^d^
**4**	IA^c^ (3)	-
**5**	4% (3)	-
**6**	IA (3)	-
**7**	2% (3)	-
**8**	IA (3)	-
**9**	32% (3)	-
**28**	4.49 ± 0.07 (4)	20% (3)
**29**	4.71 ± 0.06 (3)	18% (3)
**30**	4.87 ± 0.02 (3)	16% (3)
**31**	12% (3)	-
**32**	4.84 ± 0.10 (3)	14% (3)
**33**	5.17 ± 0.13 (4)	19% (3)
**34**	5.04 ± 0.10 (3)	5% (3)

a The estimated affinity values (p*K*_i_) for each antagonist in NanoBRET competition binding experiments with 2 μM of fluorescent antagonist **3**.

b Percentage inhibition of BRET signal with 2 μM of **3** and 100 μM of a test compound.

c IA = inactive; i.e., no inhibition of BRET signal at 100 μM. The p*K*_i_ and percentage inhibition of BRET signal experiments were carried out in membrane preparations of 1321N1 astrocytoma cells clonally expressing recombinant NanoLuc-P2Y_2_R. The final concentration of DMSO was <10%.

d Percentage inhibition of calcium mobilization induced by P2Y_2_R agonist UTPγS at 100 nM in 1321N1 astrocytoma cells expressing P2Y_2_R when treated with 10 μM of a test compound. The final concentration of DMSO was 1%. For all experiments, data points are mean values, and where appropriate ± SEM, with the number of separate experiments given in parentheses and performed in triplicate observations.

With the aim to increase the affinity of scaffold **9** through engagement of the potential P2Y_2_R basic amino acid triad (His184, Arg272, and Arg24) previously identified (Figure 2), a new series of P2Y_2_R antagonists with thiazole substituents containing terminal acidic groups were synthesized (Scheme 3). These included amino acids with varying carbon chain lengths to probe the distance required to engage the basic amino acid triad and incorporation of the tetrazole group which increased affinity ∼10-fold in the development of **1**.^19^

**Scheme 3:**
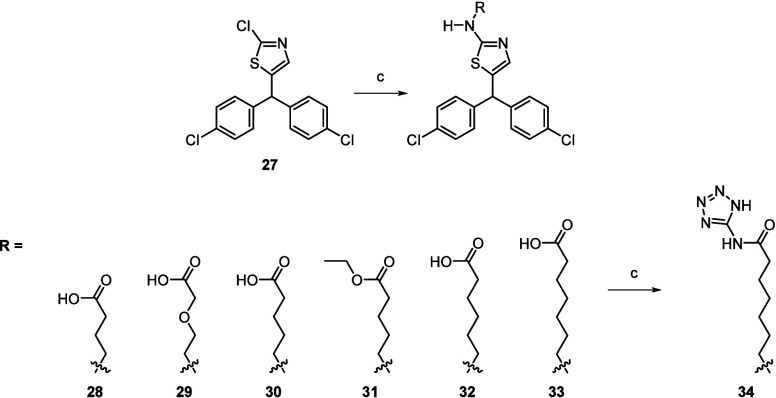
Synthesis of Compounds **28**–**34** Reagents and conditions: (a) NH_2_R, DIPEA, DMSO, 120 °C, 2–12 h (4–56%). (b) 5-Aminotetrazole monohydrate, DIPEA, PyBroP, DMF, rt, overnight (9%).

The preparation of these compounds is detailed in Scheme 3. The thiazole chlorine atom of **27** was displaced with a range of amines through nucleophilic aromatic substitution upon heating in the microwave under basic conditions to give compounds **28**-**33**. Subsequent activation with benzotriazol-1-yl-oxytripyrrolidinophosphonium hexafluorophosphate and reaction with 5-aminotetrazole was then only tolerated by compound **33** to afford the tetrazole **34**.

Compounds **28**-**34** were then evaluated using NanoBRET to assess their ability to displace **3** from NLuc-P2Y_2_R in 1321N1 cell membrane preparations, with their p*K*_i_ values reported in Table 1. The inclusion of the carboxylic acid group increased affinity, with p*K*_i_ values that could now be determined in the low micromolar range. Concurrently, protecting the carboxylic acid group with an ester, as in **31**, caused a dramatic drop in activity. Compound **29**, with the (2-aminoethoxy)acetic acid group analogous to **2**, did not demonstrate significantly improved affinity compared to **30** (*P* > 0.05 one-way ANOVA with Tukey’s multiple comparison test) which had the same carbon chain length; suggesting the ethoxy group is not making a specific interaction with the P2Y_2_R. Compound **30** had a significantly higher affinity than **28** (*** *P* < 0.001 one-way ANOVA with Tukey’s multiple comparison test), indicating that the length of the carbon chain is important for positioning the carboxylic acid group. Although not statistically significant, there was a trend for increased affinity with the presence of a longer carbon chain. Compound **33** displayed the highest affinity of the series (p*K*_i_ = 5.17 ± 0.13) which is at least a 100-fold increase on compound **9** (p*K*_i_ = ∼ 3). However, conversion of the carboxylic acid to the tetrazole in the case of **34** did not reproduce the improvement in affinity previously demonstrated for **1**.^20^ These results support the hypothesis that functionalization of the new P2Y_2_ antagonist scaffold **9** with thiazole substituents containing terminal acidic groups increases affinity through interactions with the P2Y_2_R.

We then sought to determine whether these functionalized P2Y_2_R antagonist scaffolds retained their ability to inhibit calcium mobilization induced by the P2Y_2_R agonist UTPγS in P2Y_2_R expressing 1321N1 cells. Compounds **28**-**34** were tested at 10 μM but were not fully soluble at the highest percentage of DMSO that can be tolerated by living cells. At this single point concentration of 10 μM, compounds **28**–**34** demonstrated minimal inhibition of calcium mobilization, which reflects their low affinity and insolubility (Table 1). This demonstrates the utility of NanoBRET based assays which, due to their high sensitivity, can be used to identify and monitor the binding of low affinity compounds, that offer starting points for future optimization and structure–activity relationship elucidation.^25^

These new P2Y_2_R antagonist scaffolds were then used to develop a series of fluorescent ligands for the P2Y_2_R which can be obtained in fewer synthetic steps than **3** (a 17-step synthesis with <1% overall yield).^19^ The strategy involved the incorporation of the amino acids ornithine and lysine into the P2Y_2_R antagonist scaffold, as they retain the carboxylic acid group that was crucial for affinity while the amine provides a functional handle for attachment of fluorophores. The choice of fluorophore can significantly impact the affinity and properties of a fluorescent ligand. Previous studies have demonstrated that fluorescent ligands incorporating water-soluble fluorophores, such as Alexa Fluor and sulfonated cyanine dyes, exhibit reduced affinity compared to their counterparts containing more lipophilic BODIPY and TAMRA dyes.^26−28^ These differences in affinity have been attributed to the more lipophilic fluorophores favoring association with the receptor, rather than the extracellular medium, thus presenting an opportunity to engage key residues.^27,28^ Therefore, because our P2Y_2_R antagonist scaffolds exhibited low affinity we chose to incorporate the fluorophores BODIPY 630/650-X, BODIPY FL-X, and 5-TAMRA as an opportunity to further improve affinity.

The preparation of these compounds is detailed in Scheme 4. The thiazole chlorine atom of **27** was displaced with Boc-Orn-O*t*Bu or Boc-Lys-OH upon heating in the microwave under basic conditions to yield **35** and **40**, respectively. The Boc and O*t*Bu protecting groups were then removed and the amine conjugated to the commercially available fluorophores BODIPY 630/650-X, BODIPY FL-X, and 5-TAMRA to give six fluorescent ligands (**37**-**40** and **42**-**44**) in good yields. These fluorescent ligands were then evaluated using NanoBRET saturation binding assays in membrane preparations from NLuc-P2Y_2_R 1321N1 cells.

**Scheme 4:**
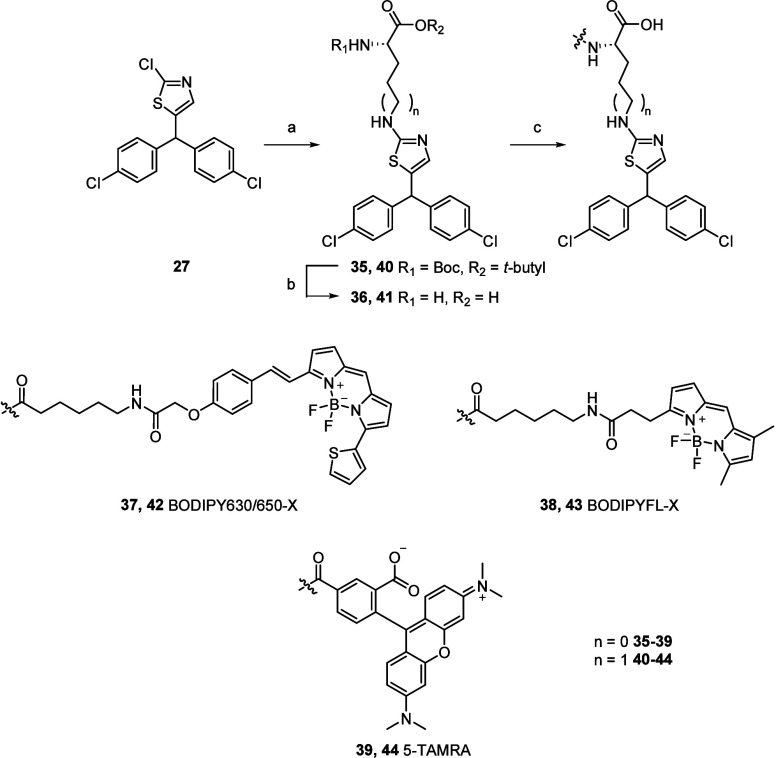
Synthesis of Compounds **35**–**44** Reagents and conditions: (a) NHR_1_R_2_, DIPEA, DMSO, 120 °C, 1 h (21%–61%) (b) TFA, DCM, 1 h, quantitative (c) BODIPY630/650–X-NHS, BODIPYFL-X-NHS or 5-TAMRA NHS ester, DIPEA, DMF, 1–4 h (25–97%).

All fluorescent ligands demonstrated some degree of specific binding, with the nonspecific component defined using a high concentration of **33** (Figure 4). However, the BODIPY FL-X containing compounds **38** and **43** had elevated levels of nonspecific binding, that could reflect the promiscuous association of these lipophilic fluorescent ligands with the membrane in close enough proximity to the NLuc-P2Y_2_R so that bystander BRET can still occur. Low levels of saturable nonspecific binding were also seen for the BODIPY 630/650-X and 5-TAMRA containing compounds **37**, **39**, **42**, and **44**, which suggests these fluorescent ligands also associate with the membrane surrounding the NLuc-P2Y_2_R but to a lesser extent. Therefore, to determine the affinity of these fluorescent ligands the nonspecific binding component was deducted from total binding and p*K*_d_ values derived where saturable specific binding was observed (Table 2).

**Table 2:** NanoBRET Affinities for P2Y_2_ Receptor Fluorescent Ligands **37**–**39** and **42**–**44**

Compound	p*K*_d_ ± (SEM)^a^
**37**	5.64 ± 0.07 (3)
**38**	ND^b^
**39**	ND
**42**	6.02 ± 0.12 (5)
**43**	ND
**44**	5.38 ± 0.19 (3)

a The estimated affinity value (p*K*_d_) for each fluorescent ligand was measured in NanoBRET saturation binding curves in membrane preparations of 1321N1 astrocytoma cells clonally expressing recombinant NanoLuc-P2Y_2_R.

b ND = not determined, i.e., not determined because binding did not saturate at 10 μM. Data points are mean values ± SEM from the number of separate experiments given in parentheses and performed in triplicate observations.

**Figure 4:**
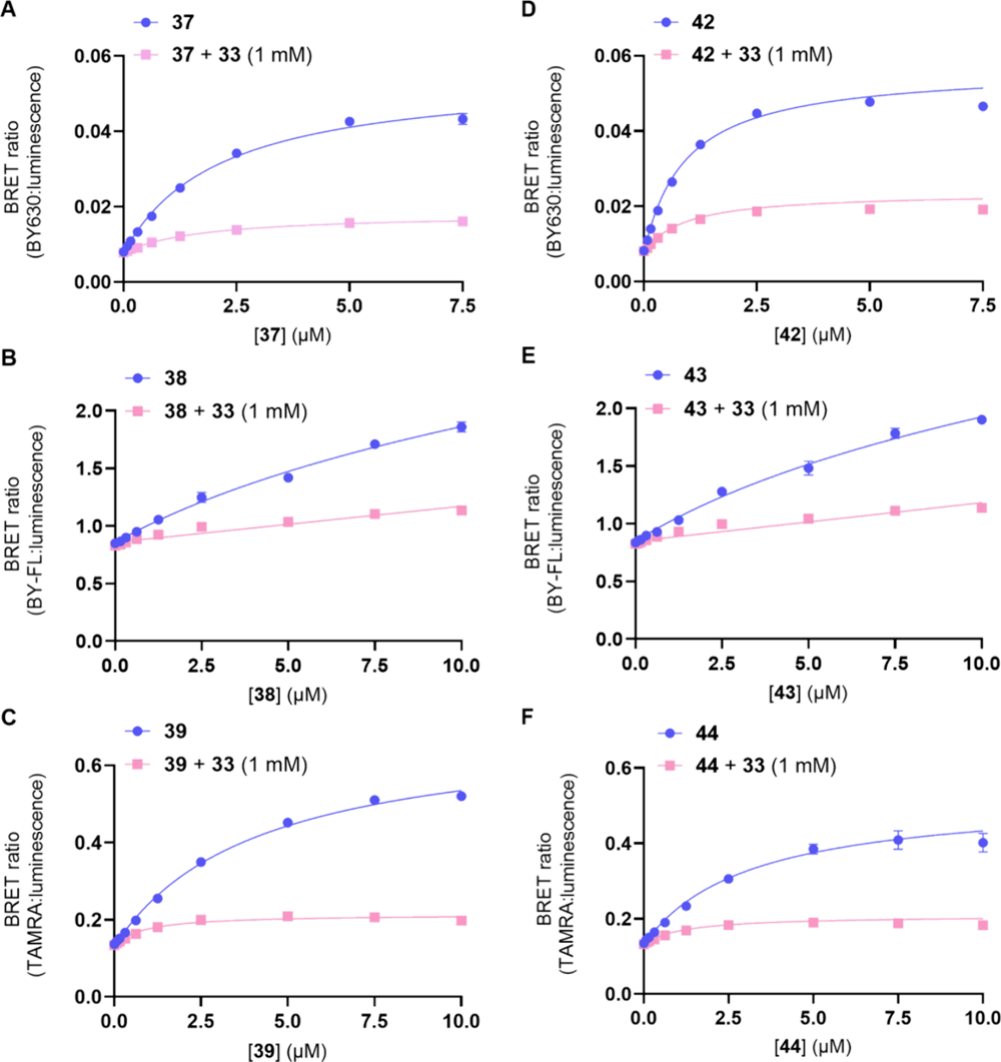
Pharmacological evaluation using NanoBRET in saturation binding assays for (A) **37**, (B) **38**, (C) **39**, (D) **42**, (E) **43**, and (F) **44** with the absence or presence of P2Y_2_R antagonist **33** at 1 mM, to determine the nonspecific binding component, in membrane preparations of 1321N1 astrocytoma cells clonally expressing recombinant NanoLuc-P2Y_2_R. The data points are the mean values of each experiment ± SEM (*n* = 3, except for **42** which is *n* = 5) and performed in triplicate observations.

Only compounds **37**, **42** and **44** demonstrated saturable specific binding (Figure 5). For both scaffolds the BODIPY 630/650-X containing ligands **37** and **42** had the highest affinity and increased affinity compared to **33**, demonstrating the ability of the fluorophore to engage with key residues in the P2Y_2_R that contribute to an improved affinity, and is consistent with previous studies.^26,29^ There was no significant difference between the affinities of **37** and **42** (*P* > 0.05 one-way ANOVA with Tukey’s multiple comparison test). However, since **44** demonstrated saturable specific binding and **39** did not, the length of the carbon chain did impact affinity of the 5-TAMRA containing fluorescent ligands.

**Figure 5:**
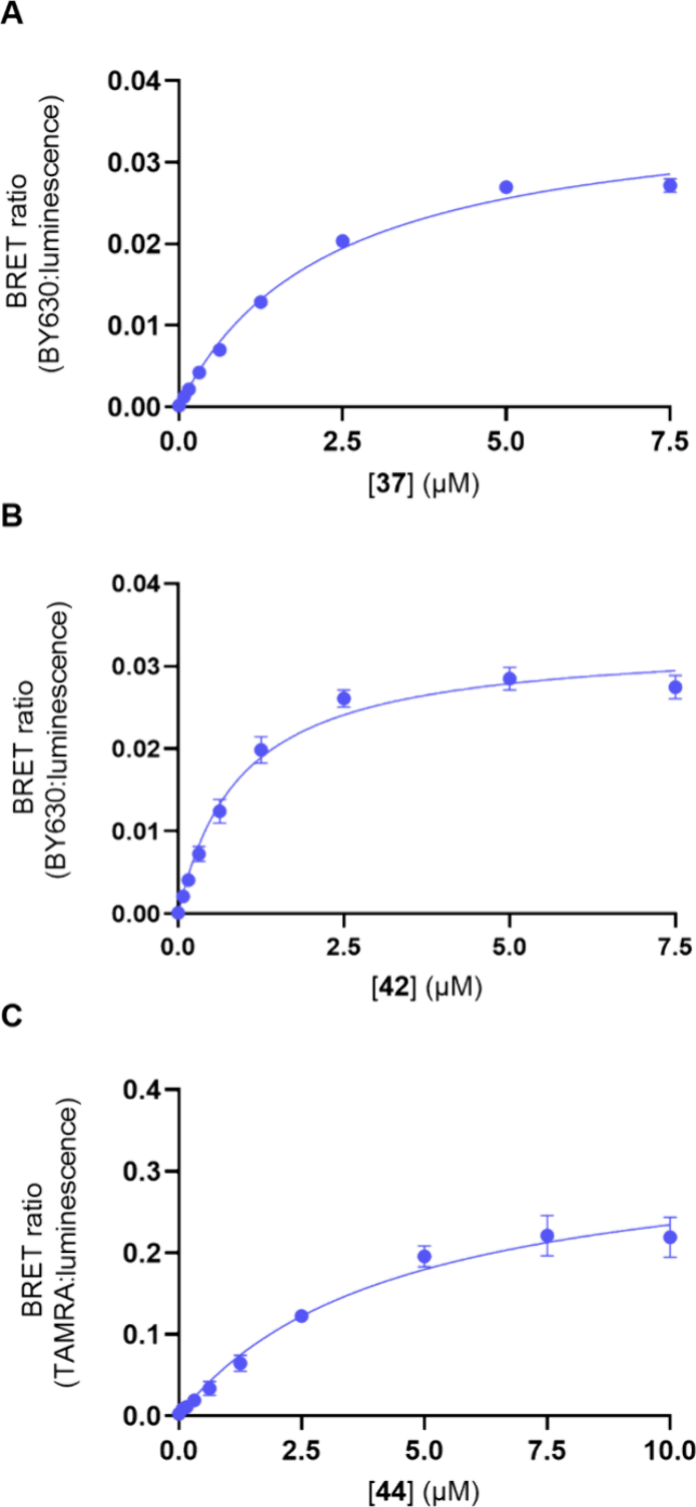
Specific binding component from NanoBRET saturation binding assays for (A) **37**, (B) **42**, and (C) **44** with the nonspecific binding component, defined with P2Y_2_R antagonist **33** at 1 mM, deducted in membrane preparations of 1321N1 astrocytoma cells clonally expressing recombinant NanoLuc-P2Y_2_R. The data points are the mean values of each experiment ± SEM (*n* = 3, except for **42** which is *n* = 5) and performed in triplicate observations.

Compound **42** had the highest affinity of all the fluorescent ligands in the micromolar range (p*K*_d_ = 6.02 ± 0.12) and a good window between the specific and nonspecific binding components. NanoBRET saturation binding assays were also carried out for compound **42** in NLuc-CXCR_4_ expressing HEK293 cell membrane preparations to investigate the contribution of bystander BRET and potential off-target effects (Supplementary Figure 2). The BRET signal for **42** in NLuc-CXCR_4_ membranes was minimal and predominantly displaceable by **33**, which suggests **42** is binding to endogenous P2Y_2_R in close enough proximity to NLuc-CXCR_4_ that bystander BRET can still occur (Supplementary Figure 2). Although not a measure of selectivity, the minimal BRET signal observed in NLuc-CXCR_4_ membranes suggests that **42** is specifically binding to the P2Y_2_R in these experiments. However, we are not able to comment on the selectivity of **42** across other P2Y receptor family members because of its low affinity and the lack of selective fluorescent ligands available for the other P2Y family members that would allow low affinity compounds to be evaluated. The affinity of fluorescent ligand **42** was also assessed using the NanoBRET competition binding assay with **3** in NLuc-P2Y_2_R membrane preparations from 1321N1 cells (Figure 6). The affinity of **42** from the NanoBRET competition binding assay (p*K*_i_ = 5.48 ± 0.30) was not significantly different from the affinity measured through the NanoBRET saturation binding assay (p*K*_i_ = 6.02 ± 0.12) (*P* > 0.05 unpaired *t* test), with complete displacement of **3** observed in a concentration-dependent manner (Figure 6).

**Figure 6:**
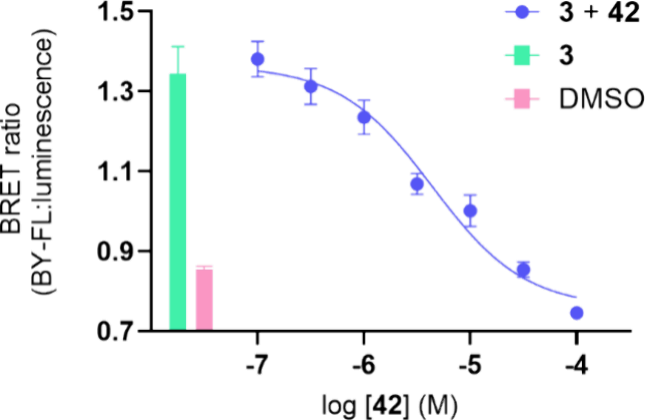
NanoBRET competition binding assay of **42** with 2 μM of fluorescent antagonist **3** in membrane preparations of 1321N1 astrocytoma cells clonally expressing recombinant NanoLuc-P2Y_2_R. The data points are the mean values of each experiment ± SEM (*n* = 5) and performed in triplicate observations.

To evaluate the utility of the new fluorescent ligand **42** we then performed NanoBRET competition binding assays with literature compound **1**, our highest affinity new P2Y_2_R antagonist **33**, and the *N*-Boc-protected precursor for **42**, compound **40** (Figure 7 and Table 3). The affinity of **1** estimated from competition binding with **42** was significantly lower (** *P* < 0.01 unpaired *t* test), with an over 1,000-fold difference, to the value determine with **3**. The affinity of compound **40** determined with **3** and **42** was also significantly different (* *P* < 0.05 unpaired *t* test), again with **42** predicting a lower affinity. The affinity of compound **2** estimated with **42** was also lower than previously determined with **3**.^19^ However, there was no significant difference between the affinities determined for compound **33** when using **3** or **42** as the fluorescent ligand (*P* > 0.05 unpaired *t* test). These results suggest that fluorescent ligand **42** might not occupy the same binding site on the P2Y_2_R to its precursor **40**, AR-C118925, and **2** but can still influence their binding, along with new P2Y_2_R antagonist scaffold **33** and fluorescent antagonist **3**.

**Figure 7:**
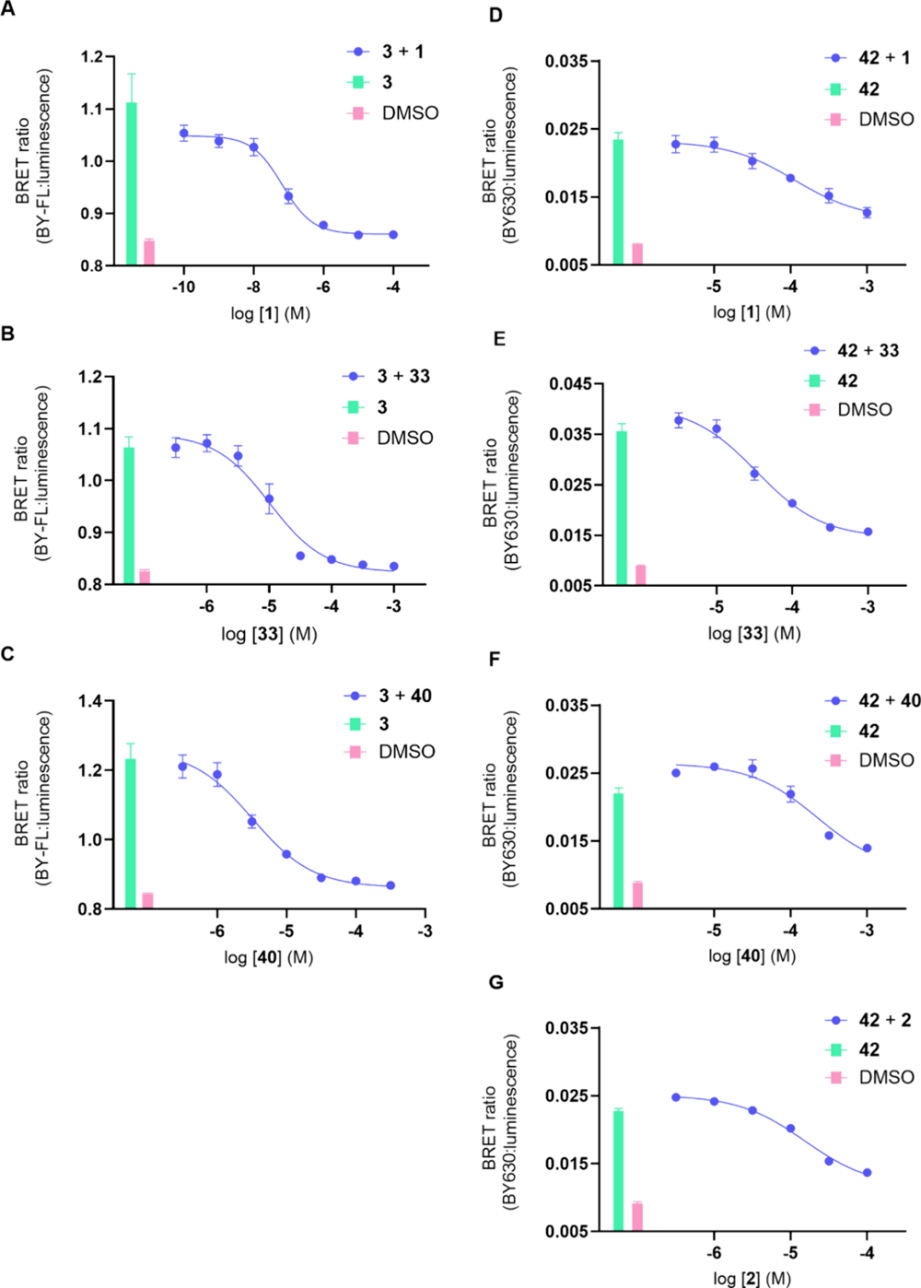
NanoBRET competition binding assays with fluorescent ligands **3** and **42** in membrane preparations of 1321N1 astrocytoma cells clonally expressing recombinant NanoLuc-P2Y_2_R. Specifically, competition binding of **1** with (A) **3** (2 μM), (D) **42** (0.75 μM); **33** with (B) **3** (2 μM), (E) **42** (1.5 μM); and **40** with either (C) **3** (2 μM) or (F) **42** (0.75 μM). The data points are the mean values of each experiment ± SEM (*n* = 3 for experiments with **3** and *n* = 5 for experiments with **42**).

**Table 3:** Comparison of Affinity Estimates for P2Y_2_ Receptor Antagonists Obtained from Competition with the Binding of Fluorescent Ligand **3** or **42**

Compound	p*K*_i_ ± (SEM) with **3**^a^	p*K*_i_ ± (SEM) with **42**^a^
**1**	7.49 ± 0.28 (3)	4.14 ± 0.07 (5)
**2**	6.56 ± 0.16 (3)^b^	5.04 ± 0.06 (5)
**33**	5.17 ± 0.13 (4)	4.86 ± 0.05 (5)
**40**	5.61 ± 0.01 (3)	3.92 ± 0.14 (5)
**42**	5.48 ± 0.30 (5)	6.02 ± 0.12 (5)^c^

a The estimated affinity value (p*K*_i_) for each antagonist was measured in NanoBRET competition binding experiments with fluorescent ligands **3** (2 μM) and **42** (0.75 μM for **1**, **2** and **40**, 1.5 μM for **33**).

b Value taken from literature.^19^

c The estimated affinity value (p*K*_d_) for **42** measured from NanoBRET saturation binding curves. All experiments were carried out in membrane preparations of 1321N1 astrocytoma cells clonally expressing recombinant NanoLuc-P2Y_2_R. Data points are mean values ± SEM from the number of separate experiments given in parentheses and performed in triplicate observations.

In conclusion, through evaluation of the binding pose of **2** in a P2Y_2_R homology model we have designed and identified the P2Y_2_R antagonist scaffold **9**, which without the tricycle ring or thiouracil retained weak affinity (p*K*_i_ = ∼ 3) for the P2Y_2_R in membranes.^19,21^ Subsequent functionalization of this P2Y_2_R antagonist scaffold with terminal carboxylic acid groups, to promote proposed engagement with a basic amino acid triad located in the P2Y_2_R binding site, improved affinity at least 100-fold in the case of compound **33** (p*K*_i_ = 5.17 ± 0.13). Compound **33** offers a starting point for further optimization to develop new P2Y_2_R antagonists and elucidate structure activity relationships.

This P2Y_2_R antagonist scaffold was then employed to develop a new series of P2Y_2_R fluorescent ligands, that were attainable in a convergent five-step synthesis. Fluorescent ligand **42** demonstrated micromolar affinity (p*K*_d_ = 6.02 ± 0.12) for the P2Y_2_R in membranes and distinct pharmacology from **3** in NanoBRET competition binding assays, which suggest it occupies a different binding site on the P2Y_2_R. The novel properties of fluorescent ligand **42** should expand the toolbox of ligands available to study the P2Y_2_R.

## Data Availability

The data that support the findings of this study are available from the corresponding author upon reasonable request.

## Supporting Information Available

Percentage inhibition of BRET signal by compound **9**, NanoBRET saturation binding for fluorescent ligand **42** in NLuc-CXCR_4_ membrane preparations, chemistry methods, and pharmacology methods (PDF)

## Abbreviations

ANOVA: analysis of variance
ATP: adenosine-5′-triphosphate
BODIPY: boron-dipyrromethene
BRET: bioluminescence resonance energy transfer
CXCR_4_: C-X-C chemokine receptor type 4
DMSO: dimethyl sulfoxide
GPCRs: G protein-coupled receptors
IC_50_: half-maximal inhibitory concentration
*K*_d_: dissociation constant of a labeled ligand–receptor complex
*K*_i_: dissociation constant of a ligand–receptor complex determined through a binding assay
NLuc: nanoluciferase
SEM: standard error of the mean
5-TAMRA: 5-carboxytetramethylrhodamine
UTP: uridine-5′-triphosphate
UTPγS: uridine-5′-(γ-thio)-triphosphate
