# Supplementary material for: Design, Synthesis, and Evaluation of a New Chemotype Fluorescent Ligand for the P2Y2 Receptor
Source: ACS Med Chem Lett. 2024 Jun 12;15(7):1127–35. doi: 10.1021/acsmedchemlett.4c00211 (PMC11247638; doi:10.1021/acsmedchemlett.4c00211)
Supplement: Supplementary file 1 — ml4c00211_si_001.pdf [file ml4c00211_si_001.pdf]

Supporting Information.

Design, Synthesis, and Evaluation of New Chemotype Fluorescent Ligand for the $P2Y_2$ Receptor

Rebecca Knight[1,2], Laura E. Kilpatrick[1,2], Stephen J. Hill[3,2], and Michael J. Stocks[1]*.

[1] Division of Biomolecular Sciences and Medicinal Chemistry, School of Pharmacy, University of Nottingham NG7 2RD, UK; [2] Centre of Membrane Proteins and Receptors (COMPARE) Universities of Birmingham and Nottingham, UK; [3] Division of Physiology, Pharmacology and Neuroscience, School of Life Sciences, University of Nottingham, NG7 2UH, UK.

*Corresponding Author: michael.stocks@nottingham.ac.uk

Contents: **Page**

Figure S1: Percentage inhibition of BRET signal by compound **9**	S2
Figure S2: NanoBRET saturation binding for fluorescent ligand **42** in NLuc-$CXCR_4$ membrane preparations	S2
Chemistry Methods	S3
Pharmacology Methods	S21

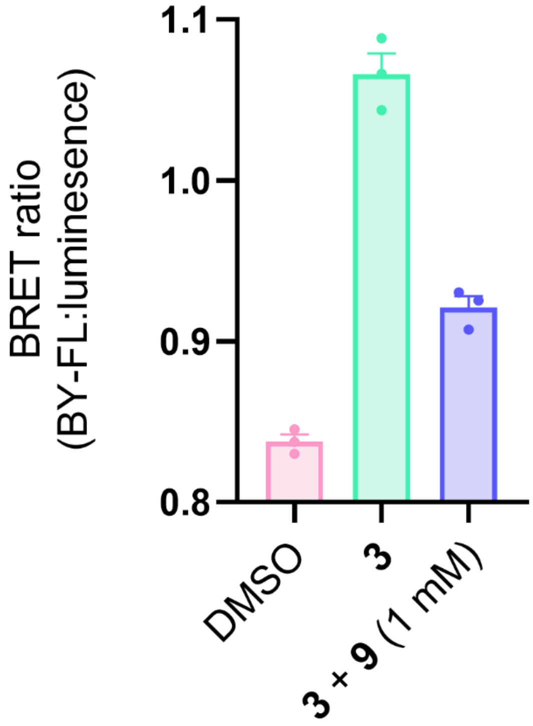

Figure S1. Inhibition of BRET signal in membrane preparations of 1321N1 astrocytoma cells clonally expressing recombinant NanoLuc-$P2Y_2R$ when treated with 2 μM of **3** and 1 mM of compound **9** (unless otherwise stated, the final concentration of DMSO was > 10%). Data points are mean values from 3 experiments (n = 3) performed in triplicate observations.

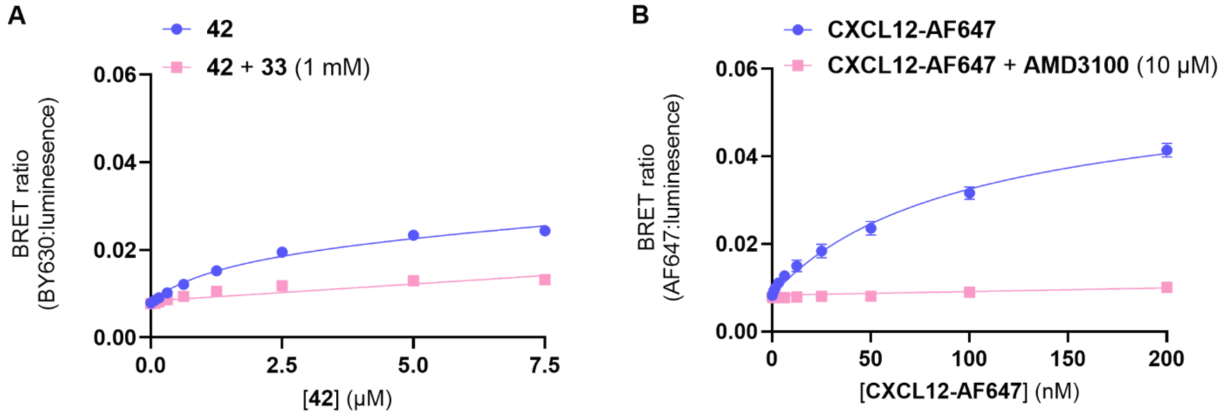

Figure S2. Pharmacological evaluation using NanoBRET in saturation binding assays for (A) **42** with absence or presence of **33** at 1 mM and (B) CXCL12-AF647 with absence or presence of AMD3100 at 10 μM, in membrane preparations of HEK239 astrocytoma cells clonally expressing recombinant NanoLuc-CXCR4. The data points are the mean values of each experiment ± SEM (n = 5).

Chemistry: Materials and Methods - No unexpected or unusually high safety hazards were encountered.

Chemicals and solvents were purchased from commercial suppliers. BODIPYFL-X-NHS ester was purchased from Molecular Probes (Thermo Fisher Scientific), while BODIPY630/650–X-NHS ester, Sulfo-Cyanine 5-NHS ester, and 5-TAMRA NHS ester were purchased from Lumiprobe (Germany).

Unless stated otherwise, all reactions were carried out at ambient temperature and compounds dried under high vacuum at rt or at 40 °C. All reactions were monitored by thin layer chromatography (TLC) using Merck Silica Gel 60 Å F254 plates with visualisation under UV light (254 nm or 366 nm) or by liquid chromatography-mass spectrometry (LC-MS). LC-MS data was collected using a Shimadzu UFLCXR high-performance liquid chromatography (HPLC) system coupled to an Applied Biosystems API 2000 LC-MS/MS with electrospray ionization (ESI+). The LC-MS UV detection was at 220 and 254 nm using a Phenomenex Gemini-NX (3 µm-110Â C18, 50 × 2mm) column at 40 °C and a flow rate of 0.5 mL/min. The LC-MS Method 1 consisted of 1 min at 5% B, 5-98% B over 2 min, 98% B for 2 min, 98-5% B over 0.5 min and then 5% for 1 min. The LC-MS Method 2 consisted of 1.5 min at 10% B, 10-98% B over 8 min, 98% B for 2 min, 98-10% B over 0.5 min and then 10% for 1 min. Solvent A contained water with 0.1% formic acid and solvent B was acetonitrile with 0.1% formic acid. Unless stated otherwise, all compounds are >95% pure.

Nuclear magnetic resonance (NMR) spectroscopy was performed using a Bruker AV(III) HD 400 NMR spectrometer equipped with a 5 mm $BBFO^+$ probe, recording ^{1}H and ^{13}C NMR at 400.25 MHz and 100.66 MHz respectively. NMR data was processed using MestReNova (version: V14.2.0-26256). Chemical shifts (δ) are quoted as values in parts per million and coupling constants (*J*) are given in hertz. Multiplicities are described using the following abbreviations: s, singlet; d, doublet; t, triplet; q, quartet; qi, quintet; sep, septet; m, multiplet; app, apparent; and br, broad.

Flash column chromatography was performed using silica gel 60, 230-400 mesh particle size (Sigma Aldrich). Automated flash column normal phase chromatography was performed on a Biotage Isolera One system (ISO-1SV) equipped with a UV detector (200-400 nm) using silica high performance (50 µm) cartridges. Methods were developed and run using Biotage Isolera (version: 3.3.0) software. Automated flash column reverse-phase chromatography was performed on a Interchim Puriflash 4100 system (PF4100-250) equipped with a dual wavelength DAD UV detector (200-600 nm) using C18-HP (30 µm) cartridges. Methods were developed and run using Interchim Flash (version: V5.1c.09) software.

RP-HPLC was performed on a Waters 515 LC system and monitored using a Waters 996 photodiode array detector at 190–800 nm or on a Shimadzu Nexera XR system using a SPD-M40 photodiode array detector at 190–800 nm. The spectra were analysed using either Millenium 32 software or LabSolutions. Solvent A contained water with 0.1% formic acid and solvent B was acetonitrile with 0.1% formic acid. Semipreparative HPLC was performed using a Phenomenex Gemini-NX (5 µm, 110 Å, C18, 250 x 10 mm) column at ambient temperature with a flow rate of 5 mL/min. Analytical HPLC was performed using a YMC-Pack Pro C8 (3 µm, 12 nM, C8, 150 x 4.6 mm) column at ambient temperature with a flow rate of 1 mL/min. **Method A** ran for 1 min at 40% B, then 40–45% B over 4 min, 45–50% B over 20 min, 50–95% B over 2 mins, and then 95–40% B over 1 min. **Method B** ran for 1 min at 40% B, then 40–70% B over 9 min, 70–90% B over 1 min, and then 90–40% B over 1 min. **Method C** ran for 1 min at 10% B, then 10–90% B over 13 min, 90% B for 1 min, and then 90–10% B over 1 min. **Method D** ran for 1 min at 20% B, then 20–90% B over 13 min, 90% B for 1 min, and then 90–20% B over 1 min. **Method E** ran for 1 min at 45% B, then 45–60% B over 1 min, 60–90% B for 12 min, 90% B for 1 min, and then 90–45% B over 1 min. **Method F** ran for 1 min at 10% B, then 10–95% B over 23 mins,

95% B for 2 mins, then 95–10% B over 1 min and then 10% B for 3 mins. High-resolution mass spectrometry (HRMS) was achieved using a Bruker microTOF II MS with electrospray ionization (TOF ESI+).

General Procedure 1: Grignard reaction.

To a stirred solution of the respective ester or Weinreb amide (1 equiv.) in anhydrous tetrahydrofuran (THF) (0.1 M) was dropwise added 4-chlorophenylmagnesium bromide (4 equiv.) at -78 °C under N_2. This was stirred overnight and allowed to warm to room temperature (rt). For reactions with an ester, the reaction mixture (RM) was quenched with water, concentrated *in vacuo*, filtered through celite, and washed with ethyl acetate. For reactions with a Weinreb amide the RM was quenched with water, treated with 2 M hydrochloric acid (HCl), and then concentrated *in vacuo*. In both cases, the organic components were then extracted with ethyl acetate and the combined organic layers washed with sat. brine, dried over anhydrous Na_2SO_4, filtered, and concentrated *in vacuo*. Purification for the ester product was achieved by reverse-phase flash chromatography using a gradient of 10–90% acetonitrile/water followed by lyophilisation. Purification for the Weinreb amide product was achieved by normal-phase flash chromatography using a gradient of 0–5% ethyl acetate: industrial methylated spirit (3:1)/cyclohexane.

General Procedure 2: Triethylsilane mediated reduction of tertiary alcohols.

To a stirred solution of the respective tertiary alcohol (1 equiv.) in anhydrous dichloromethane (DCM) (0.05 M) was added triethylsilane (5 equiv.) under N_2. This was cooled to 0 °C for the dropwise addition of trifluoroacetic acid (TFA) (20 equiv.) and then stirred for 30 mins at rt. The RM was quenched with sat. aq. $NaHCO_3$, the organic components extracted with DCM, and then the combined organic layers were washed with sat. brine, dried over anhydrous Na_2SO_4, filtered, and concentrated *in vacuo*. Purification was achieved by reverse-phase flash chromatography using a gradient of 10-90% acetonitrile/water followed by lyophilisation.

General Procedure 3: Substitution of 2-chlorothiazoles to 2-aminothiazoles.

To a solution of the respective 2-chlorothiazole (1 equiv.) in dimethyl sulfoxide (DMSO) (0.1 M) was added the chosen amine (4 equiv.) and DIPEA (10 equiv.) or triethylamine (10 equiv.) for 2-ethoxyethylamine. The stirred solution was heated in a microwave reactor at 120-150 °C for 0.5–16h and then concentrated *in vacuo*.

General Procedure 4: Synthesis of fluorescent conjugates

The deprotected amine (1 equiv.) was dissolved in DMF (4 mM), added to a solution of BODIPYFL-X-NHS (1 equiv.), BODIPY630/650–X-NHS (1 equiv.), Sulfo-Cyanine 5-NHS ester (1 equiv.), or 5-TAMRA NHS ester (1 equiv.) in DMF (13.5 mM), and treated with DIPEA (100 equiv.) to react in the absence of light for 2-16 h.

5-((4-chlorophenyl)(2-((2-ethoxyethyl)amino)thiazol-4-yl)methyl)-1-methyl-4-thioxo-3,4-dihydropyrimidin-2(1*H*)-one (4)

To a stirred solution of **5** (1 equiv., 240 mg, 0.57 mmol) in anhydrous 1,4-dioxane (4 mL) was added Lawesson's reagent (2 equiv., 461 mg, 1.14 mmol) under N_2 and then refluxed (120 °C) for 48 hours. This was then concentrated *in vacuo* directly onto silica and purified by normal-phase flash chromatography using a gradient of 0–30% ethyl acetate: industrial methylated spirit (3:1)/cyclohexane followed

by reverse-phase flash chromatography using a gradient of 10–90% acetonitrile/water to afford **4** as an orange oil (9 mg, 0.02 mmol, 4%).

LC-MS (ESI+) *m/z* calc. for $C_{19}H_{22}ClN_4O_2S_2$ $[M(^{35}Cl)+H]^+$ = 437.1, found = 436.7, t_R = 2.58 min (Method 1).
HRMS (TOF ESI+) *m/z* calc. for $C_{19}H_{21}ClN_4NaO_2S_2$ $[M+Na]^+$ = 459.0687, found = 459.0706; *m/z* calc. for $C_{19}H_{22}ClN_4O_2S_2$ $[M+H]^+$ = 437.0867, found = 437.0879.
^{1}H NMR (400 MHz, $CDCl_3$) δ 9.92 (s, 1H), 7.30 – 7.27 (m, 2H), 7.24 – 7.20 (m, 2H), 7.16 (s, 1H), 6.07 (s, 1H), 5.84 (s, 1H), 3.65 (t, J = 5.1 Hz, 2H), 3.55 (q, J = 7.0 Hz, 2H), 3.43 (m, 2H), 3.38 (s, 3H), 1.23 (t, J = 7.0 Hz, 3H) (N.B. 2-ethoxyethan-1-aminyl -N**H** not observed).
^{13}C NMR (101 MHz, $CDCl_3$) δ 189.07, 170.24, 148.1, 141.48, 139.17, 132.71, 129.96, 128.57, 123.72, 104.94, 68.29, 66.65, 46.48, 45.68, 36.91, 15.12.

5-((4-chlorophenyl)(2-((2-ethoxyethyl)amino)thiazol-4-yl)methyl)-1-methylpyrimidine-2,4(1*H*,3*H*)-dione (5).

Following general procedure 3, **16** (84 mg, 0.23 mmol) was converted to **5** and purified by reverse-phase flash chromatography using a gradient of 10–90% acetonitrile/water followed by lyophilisation to afford a white solid (25 mg, 0.06 mmol, 26%).

LC-MS (ESI+) *m/z* calc. for $C_{19}H_{22}ClN_4O_3S$ $[M(^{35}Cl)+H]^+$ = 421.1, found = 421.2, t_R = 2.36 min (Method 1).
HRMS (TOF ESI+) *m/z* calc. for $C_{19}H_{22}ClN_4O_3S$ $[M+H]^+$ = 421.1096, found = 421.1094; *m/z* calc. for $C_{19}H_{21}ClN_4NaO_3S$ $[M+Na]^+$ = 443.0915, found = 443.0900.
^{1}H NMR (400 MHz, $CDCl_3$) δ 8.59 (s, 1H), 7.35 – 7.25 (m, 3H), 7.28 – 7.20 (m, 2H), 6.07 (d, *J* = 0.8 Hz, 1H), 5.59 (s, 1H), 5.32 (s, 1H), 3.65 (t, *J* = 5.2 Hz, 2H), 3.55 (q, *J* = 7.0 Hz, 2H), 3.41 (q, *J* = 4.9 Hz, 2H), 3.35 (s, 3H), 1.23 (t, *J* = 7.0 Hz, 3H).
^{13}C NMR (101 MHz, $CDCl_3$) δ 170.28, 162.93, 151.84, 150.73, 143.71, 139.26, 132.73, 129.85, 128.62, 115.85, 104.48, 68.33, 66.63, 45.62, 43.55, 36.21, 15.12.

4-(*bis*(4-Chlorophenyl)methyl)-*N*-(2-ethoxyethyl)thiazol-2-amine (6).

Following general procedure 3, **25** (75 mg, 0.21 mmol) was converted to **6** and purified by reverse-phase flash chromatography using a gradient of 10–90% acetonitrile/water followed by lyophilisation to afford an orange oil (46 mg, 0.11 mmol, 53%).

LC-MS (ESI+) *m/z* calc. for $C_{20}H_{21}Cl_2N_2OS$ $[M+H]^+$ = 407.1, found = 407.3, t_R = 3.28 min (Method 1).
HRMS (TOF ESI+) *m/z* calc. $C_{20}H_{21}Cl_2N_2OS$ $[M(^{35}Cl)+H]^+$ = 407.0746, found = 407.0744.
^{1}H NMR (400 MHz, $CDCl_3$) δ 7.32 – 7.23 (m, 4H), 7.16 – 7.07 (m, 4H), 5.87 (d, *J* = 1.0 Hz, 1H), 5.53 (s, 1H), 5.33 (d, *J* = 5.1 Hz, 2H), 3.65 – 3.58 (m, 2H), 3.52 (q, *J* = 7.0 Hz, 2H), 3.38 (q, *J* = 4.8 Hz, 2H), 1.21 (t, *J* = 7.0 Hz, 3H).
^{13}C NMR (101 MHz, $CDCl_3$) δ 169.89, 154.18, 140.77, 132.52, 130.38, 128.55, 104.50, 68.38, 66.59, 52.60, 45.58, 15.12.

5-((4-chlorophenyl)(2-((2-ethoxyethyl)amino)thiazol-5-yl)methyl)-1-methyl-4-thioxo-3,4-dihydropyrimidin-2(1*H*)-one (7).

To a stirred solution of **8** (1 equiv., 79 mg, 0.19 mmol) in anhydrous 1,4-dioxane (4 mL) was added Lawesson's reagent (2 equiv., 154 mg, 0.38 mmol) under N_2 and then refluxed (120 °C) overnight. This was then concentrated *in vacuo* directly onto silica and purified by normal-phase flash chromatography

using a gradient of 10–25% ethyl acetate: industrial methylated spirit (3:1)/cyclohexane to afford **7** as a yellow solid (36 mg, 0.08 mmol, 43%).

LC-MS (ESI+) *m/z* calc. for $C_{19}H_{22}ClN_4O_2S_2$ $[M(^{37}Cl)+H]^+$ = 439.1, found = 438.9, t_R = 2.67 min (Method 1).
HRMS (TOF ESI+) *m/z* calc. for $C_{19}H_{21}ClN_4NaO_2S_2$ $[M+Na]^+$ = 459.0687, found = 459.0697.
^{1}H NMR (400 MHz, DMSO) δ 12.82 (s, 1H), 12.16 (d, *J* = 2.6 Hz, 1H), 7.44 – 7.35 (m, 2H), 7.28 – 7.19 (m, 3H), 6.31 (d, *J* = 1.1 Hz, 1H), 6.00 (s, 1H), 3.94 – 3.77 (m, 2H), 3.57 (tt, *J* = 9.9, 5.7 Hz, 2H), 3.48 – 3.34 (m, 2H), 3.27 (s, 3H), 1.09 (t, *J* = 7.0 Hz, 3H).
^{13}C NMR (101 MHz, DMSO) δ 189.32, 162.17, 148.88, 143.21, 138.81, 132.15, 131.75, 130.76, 128.97, 120.65, 114.64, 66.99, 66.23, 44.28, 40.87, 36.84, 15.52.

5-((4-chlorophenyl)(2-((2-ethoxyethyl)amino)thiazol-5-yl)methyl)-1-methylpyrimidine-2,4(1*H*,3*H*)-dione (8).

Following general procedure 3, **23** (208 mg, 0.56 mmol) was converted to **8** and purified by reverse-phase flash chromatography using a gradient of 10–90% acetonitrile/water followed by lyophilisation to afford a white solid (105 mg, 0.25 mmol, 45%).

LC-MS (ESI+) *m/z* calc. for $C_{19}H_{22}ClN_4O_3S$ $[M(^{35}Cl)+H]^+$ = 421.1, found = 421.1, t_R = 2.60 min (Method 1).
HRMS (TOF ESI+) *m/z* calc. for $C_{19}H_{22}ClN_4O_3S$ $[M+H]^+$ = 421.1096, found = 421.1094; *m/z* calc. for $C_{19}H_{21}ClN_4NaO_3S$ $[M+Na]^+$ = 443.0915, found = 443.0900.
^{1}H NMR (400 MHz, DMSO-*d6*) δ 12.21 (s, 1H), 11.40 (s, 1H), 7.44 – 7.35 (m, 2H), 7.28 – 7.19 (m, 2H), 7.09 (s, 1H), 6.35 (d, *J* = 1.7 Hz, 1H), 5.41 (s, 1H), 3.87 (t, *J* = 5.9 Hz, 2H), 3.59 – 3.44 (m, 2H), 3.20 (s, 3H), 1.08 (t, *J* = 7.0 Hz, 3H) (N.B. -OC**H**2CH3 under water peak but visible).
^{13}C NMR (101 MHz, DMSO-*d6*) δ 163.36, 162.07, 151.38, 144.90, 138.90, 132.20, 131.47, 130.61, 129.07, 114.43, 112.53, 67.17, 66.22, 44.28, 37.61, 35.93, 15.41.

5-(*bis*(4-Chlorophenyl)methyl)-*N*-(2-ethoxyethyl)thiazol-2-amine (9).

Following general procedure 3, **27** (60 mg, 0.17 mmol) was converted to **9** and purified by reverse-phase flash chromatography using a gradient of 10–90% acetonitrile/water followed by lyophilisation to afford an yellow oil (39 mg, 0.096 mmol, 57%).

LC-MS (ESI+) *m/z* calc. for $C_{20}H_{21}Cl_2N_2OS$ $[M(^{35}Cl)+H]^+$ = 407.1, found = 407.9, t_R = 3.36 min (Method 1).
HRMS (TOF ESI+) *m/z* calc. $C_{20}H_{21}Cl_2N_2OS$ $[M+H]^+$ = 407.0746, found = 407.0748. ^{1}H NMR (400 MHz, $CDCl_3$) δ 7.32 – 7.29 (m, 4H), 7.19 – 7.11 (m, 4H), 6.61 (d, *J* = 1.2 Hz, 1H), 5.41 (s, 1H), 5.32 (s, 1H), 3.62 (t, *J* = 5.6, 4.6 Hz, 2H), 3.53 (q, *J* = 7.0 Hz, 2H), 3.44 (t, *J* = 5.2 Hz, 2H), 1.22 (t, *J* = 7.0 Hz, 3H).
^{13}C NMR (101 MHz, $CDCl_3$) δ 169.93, 141.08, 137.72, 132.90, 129.96, 128.72, 128.41, 68.49, 66.54, 48.63, 45.24, 15.12.

2-chlorothiazole-4-carboxylic acid (11).

To a stirred solution ethyl 2-chlorothiazole-4-carboxylate (1 equiv., 2.40 g, 12.52 mmol) in THF (35 mL) was added NaOH (1.2 equiv., 600 mg, 15.00 mmol) and water (35 mL). This was stirred overnight and then diluted with water (35 mL) before concentration *in vacuo*. The pH of the RM was adjusted to ~2 with 2 M HCl and the organic components extracted with ethyl acetate (3 x 40 mL), the combined

organic layers were washed with sat. brine (40 mL), dried over anhydrous Na_2SO_4, filtered, and concentrated *in vacuo* to afford **11** as a white solid (2.00 g, 12.23 mmol, 98%).

LC-MS (ESI+) *m/z* calc. for $C_4H_3ClNO_2S$ $[M(^{35}Cl)+H]^+$ = 164.0, found = 164.0, t_R = 1.42 min (Method 1).
HRMS (TOF ESI+) *m/z* calc. $C_4H_2ClNNaO_2S$ $[M+Na]^+$ = 185.9387, found = 185.9386.
^{1}H NMR (400 MHz, DMSO-d_6) δ 13.42 (s, 1H), 8.41 (s, 1H).
^{13}C NMR (101 MHz, DMSO-d_6) δ 161.45, 151.49, 146.00, 132.09.

2-chloro-*N*-methoxy-*N*-methylthiazole-4-carboxamide (12).

To a solution of **11** (1 equiv., 2.00 g, 12.23 mmol) in anhydrous THF (35 mL) at 0 °C was added oxalyl chloride (3 equiv., 3.1 mL, 36.69 mmol) and DMF (0.05 equiv., 0.61 mmol, one drop) under N_2. This was stirred for 5 hours at rt and then concentrated *in vacuo*. The yellow solid was dissolved in anhydrous DCM (25 mL) and to the solution at 0 °C was added DIPEA (3 equiv., 6.4 mL, 36.6 mmol) and *N*,*O*-dimethylhydroxylamine-HCl (1 equiv., 1.2 g, 12.2 mmol). The RM was stirred overnight and then diluted with 1 M KH_2PO_4 (35 mL) before extraction with DCM (3 x 50 mL). The combined organic layers were washed with sat. brine (50 mL), dried over anhydrous Na_2SO_4, filtered, and concentrated *in vacuo* to afford **12** as a brown oil (2.50 g, 12.10 mmol, 99%).

LC-MS (ESI+) *m/z* calc. for $C_6H_8ClN_2O_2S$ $[M(^{35}Cl)+H]^+$ = 207.0, found = 207.1, t_R = 2.37 min (Method 1).
HRMS (TOF ESI+) *m/z* calc. $C_6H_8ClN_2O_2S$ $[M+H]^+$ = 206.9990, found = 206.9993; *m/z* calc. $C_6H_7ClN_2NaO_2S$ $[M+Na]^+$ = 228.9809, found = 228.9810.
^{1}H NMR (400 MHz, $CDCl_3$) δ 7.94 (s, 1H), 3.80 (s, 3H), 3.42 (s, 3H).
^{13}C NMR (101 MHz, $CDCl_3$) δ 161.76, 151.07, 147.28, 126.96, 61.71, 53.60.

(4-chlorophenyl)(2-chlorothiazol-4-yl)methanone (13). Following general procedure 1, **12** (2.5 g, 12.10 mmol) was converted to **13** which was isolated as a white solid (1.95 g, 7.55 mmol, 62%).

LC-MS (ESI+) *m/z* calc. for $C_{10}H_6Cl_2NOS$ $[M(^{35}Cl)+H]^+$ = 258.0, found = 257.8, t_R = 2.98 min (Method 1).
HRMS (TOF ESI+) *m/z* calc. $C_{10}H_5Cl_2NNaOS$ $[M+Na]^+$ = 279.9361, found = 279.9361.
^{1}H NMR (400 MHz, $CDCl_3$) δ 8.33 (s, 1H), 8.25 – 8.19 (m, 2H), 7.54 – 7.45 (m, 2H).
^{13}C NMR (101 MHz, $CDCl_3$) δ 184.24, 152.39, 151.86, 139.84, 134.72, 132.05, 129.87, 128.72.

(4-chlorophenyl)(2-chlorothiazol-4-yl)(2,4-di-*tert*-butoxypyrimidin-5-yl)methanol (14).

To stirred solution of 5-bromo-2,4-di-*tert*-butoxypyrimidine (1 equiv., 1.30g, 4.30 mmol) in anhydrous, unstabilized THF (34 mL) cooled to -78 °C was added *n*-butyllithium (1.5 equiv., 2.5 M in hexane, 2.6 mL, 6.46 mmol) dropwise under N_2. This was stirred for 15 minutes before the addition of the corresponding ketone (1 equiv., 1.11 g, 4.30 mmol)), which had been dissolved in anhydrous, unstabilized THF (7 mL) under N_2. This was stirred for 15 min at -78 °C and then allowed to warm to rt over 1 hour before being quenched with sat. aq. NH_4Cl (20 mL) and water (20 mL). The organic components were extracted with ethyl acetate (3 x 50 mL and the combined organic layers washed with sat. brine (20 mL), dried over anhydrous Na_2SO_4, filtered, and concentrated *in vacuo*. Purification was achieved by normal-phase flash chromatography using a gradient of 0-20% ethyl acetate/cyclohexane to afford a brown crystalline solid (355 mg, 0.74 mmol, 17%).

LC-MS (ESI+) *m/z* calc. for $C_{22}H_{26}Cl_2N_3O_3S$ $[M(^{35}Cl)+H]^+$ = 482.1, found = 482.3, t_R = 3.40 min (Method 1).
HRMS (TOF ESI+) *m/z* calc. for $C_{22}H_{26}Cl_2N_3O_3S$ $[M+H]^+$ = 482.1066, found = 482.1061; *m/z* calc. $C_{22}H_{25}Cl_2N_3NaO_3S$ $[M+Na]^+$ = 504.0886, found = 504.0892.
^{1}H NMR (400 MHz, CDCl3) δ 7.69 (s, 1H), 7.40 – 7.36 (m, 2H), 7.35 – 7.30 (m, 2H), 7.08 (s, 1H), 4.47 (s, 1H), 1.62 (s, 9H), 1.40 (s, 9H).
^{13}C NMR (101 MHz, CDCl3) δ 167.16, 163.70, 158.15, 157.03, 151.56, 142.39, 133.56, 128.19, 128.15, 119.08, 118.34, 83.65, 80.75, 77.35, 77.03, 76.83, 76.72, 28.36, 28.32.

5-((4-chlorophenyl)(2-chlorothiazol-4-yl)methyl)pyrimidine-2,4(1H,3H)-dione (15).

Following general procedure 2, **14** (355 mg, 0.74 mmol) was converted to **15** which was isolated as a white solid (188 mg, 0.53 mmol, 71%).

LC-MS (ESI+) *m/z* calc. for $C_{14}H_{10}Cl_2N_3O_2S$ $[M(^{35}Cl)+H]^+$ = 354.0, found = 354.1, t_R = 2.60 min (Method 1).
HRMS (TOF ESI+) *m/z* calc. for $C_{14}H_9Cl_2N_3NaO_2S$ $[M+Na]^+$ = 375.9685, found = 375.9688.
^{1}H NMR (400 MHz, DMSO-d_6) δ 11.21 (s, 1H), 10.76 (d, 1H), 7.40 – 7.34 (m, 3H), 7.28 – 7.22 (m, 2H), 6.93 (d, *J* = 5.7 Hz, 1H), 5.37 (s, 1H).
^{13}C NMR (101 MHz, DMSO-d_6) δ 163.91, 155.27, 151.40, 150.73, 140.30, 140.07, 131.94, 130.78, 128.89, 120.45, 113.39, 43.64.

5-((4-chlorophenyl)(2-chlorothiazol-4-yl)methyl)-1-methylpyrimidine-2,4(1*H*,3*H*)-dione (16).

To a stirred solution of **15** (1 equiv., 188 mg, 0.53 mmol) in anhydrous DCE (5 mL) was added BSTFA (4 equiv., 0.6 mL, 2.12 mmol) under N_2. This was refluxed overnight and then cooled before the addition of iodomethane (20 equiv., 0.7 mL, 10.60 mmol). The RM was then heated to 50 °C for 18 hours. The organic components were extracted with DCM (3 x 5 mL) and the combined organic layers washed with sat. brine (5 mL), dried over anhydrous Na_2SO_4, filtered, and concentrated *in vacuo*. Purification was achieved by normal-phase flash chromatography using a gradient of 10–80% ethyl acetate/cyclohexane to afford **16** as a yellow solid (118 mg, 0.32 mmol, 61%).

LC-MS (ESI+) *m/z* calc. for $C_{15}H_{12}Cl_2N_3O_2S$ $[M(^{35}Cl)+H]^+$ = 368.0, found = 368.0, t_R = 2.78 min (Method 1).
HRMS (TOF ESI+) *m/z* calc. for $C_{15}H_{12}Cl_2N_3O_2S$ $[M+H]^+$ = 368.0022, found = 368.0011; *m/z* calc. for $C_{15}H_{11}Cl_2N_3NaO_2S$ $[M+Na]^+$ = 389.9841, found = 389.9841.
^{1}H NMR (400 MHz, $CDCl_3$) δ 8.81 (s, 1H), 7.36 – 7.28 (m, 2H), 7.27 – 7.22 (m, 2H), 7.06 (s, 1H), 6.94 (s, 1H), 5.49 (s, 1H), 3.36 (s, 3H).
^{13}C NMR (101 MHz, $CDCl_3$) δ 162.87, 154.34, 152.17, 150.59, 143.93, 138.31, 133.26, 129.84, 128.97, 118.68, 115.34, 43.37, 36.32.

2-chlorothiazole-5-carboxylic acid (18).

To a stirred solution ethyl 2-chlorothiazole-5-carboxylate (1 equiv., 5.00 g, 26.09 mmol) in ethanol (60 mL) was added NaOH (1.2 equiv., 1.25 g, 31.25 mmol) and water (30 mL). This was stirred overnight and then diluted with water (30 mL) before concentration *in vacuo*. The pH of the RM was adjusted to ~2 with 2 M HCl and the organic components extracted with ethyl acetate (3 x 60 mL). The combined organic layers were washed with sat. brine (60 mL), dried over anhydrous Na_2SO_4, filtered, and concentrated *in vacuo* to afford **18** as a white solid (4.16 g, 25.43 mmol, 97%).

LC-MS (ESI+) *m/z* calc. for $C_4H_3ClNO_2S$ $[M(^{35}Cl)+H]^+$ = 164.0, found = not observed, t_R = 2.48 min (Method 1).
HRMS (TOF ESI+) *m/z* calc. $C_4H_3ClNO_2S$ $[M+H]^+$ = 163.9568, found = 163.9572.
^{1}H NMR (400 MHz, DMSO-d_6) δ 13.92 (s, 1H), 8.23 (s, 1H).
^{13}C NMR (101 MHz, DMSO-d_6) δ 161.46, 155.70, 146.94, 133.49.

2-chloro-*N*-methoxy-*N*-methylthiazole-5-carboxamide (19).

To a solution of **18** (1 equiv., 4.12 g, 25.19 mmol) in anhydrous DCM (25 mL) at 0 °C was added oxalyl chloride (3 equiv., 6.40 mL, 75.57 mmol) and DMF (0.05 equiv., 1.26 mmol, 0.10 mL) under N_2. This was stirred for 5 hours at rt and then concentrated *in vacuo* to a yellow solid. This was dissolved in anhydrous DCM (25 mL) and to the solution at 0 °C was added DIPEA (3 equiv., 13.2 mL, 75.6 mmol) and *N*,*O*-dimethylhydroxylamine-HCl (1 equiv., 2.46 g, 25.2 mmol). The RM was stirred overnight and then diluted with 1 M KH_2PO_4 (25 mL). The organic components were extracted with DCM (3 x 50 mL) and the combined organic layers were washed with sat. brine (50 mL), dried over anhydrous Na_2SO_4, filtered, and concentrated *in vacuo* to afford **19** as a brown oil (5.12 g, 24.78 mmol, 98%).

LC-MS (ESI+) *m/z* calc. for $C_6H_8ClN_2O_2S$ $[M(^{35}Cl)+H]^+$ = 207.0, found = 207.0, t_R = 2.75 min (Method 1).
HRMS (TOF ESI+) *m/z* calc. $C_6H_8ClN_2O_2S$ $[M+H]^+$ = 206.9990, found = 206.9993; *m/z* calc. $C_6H_7ClN_2NaO_2S$ $[M+Na]^+$ = 228.9809, found = 228.9803.
^{1}H NMR (400 MHz, $CDCl_3$) δ 8.32 (s, 1H), 3.79 (s, 3H), 3.37 (s, 3H).
^{13}C NMR (101 MHz, $CDCl_3$) δ 160.03, 157.99, 147.71, 129.56, 61.81, 32.66.

(4-chlorophenyl)(2-chlorothiazol-5-yl)methanone (20).

Following general procedure 1, **19** (5.30 g, 25.65 mmol) was converted to **20** which was isolated as a yellow powder (2.66g, 10.31 mmol, 40%).

LC-MS (ESI+) *m/z* calc. for $C_{10}H_6Cl_2NOS$ $[M(^{35}Cl)+H]^+$ = 258.0, found = 257.8, t_R = 3.21 min (Method 1).
HRMS (TOF ESI+) *m/z* calc. $C_{10}H_6Cl_2NOS$ $[M+H]^+$ = 257.9542, found = 257.9538.
^{1}H NMR (400 MHz, $CDCl_3$) δ 8.01 (s, 1H), 7.89 – 7.79 (m, 2H), 7.60 – 7.50 (m, 2H).
^{13}C NMR (101 MHz, $CDCl_3$) δ 185.04, 159.11, 146.93, 141.15, 139.99, 135.06, 130.42, 129.30.

(4-chlorophenyl)(2-chlorothiazol-5-yl)(2,4-di-*tert*-butoxypyrimidin-5-yl)methanol (21).

To stirred solution of 5-bromo-2,4-di-*tert*-butoxypyrimidine (1 equiv., 117m g, 0.38 mmol) in anhydrous, unstabilized THF (3 mL) cooled to -78 °C was added *n*-butyllithium (2 equiv., 2.5 M in hexane, 0.3 mL, 0.76 mmol) dropwise under N_2. This was stirred for 15 minutes before the addition of the corresponding ketone (1 equiv., 100 mg, 0.38 mmol), which had been dissolved in anhydrous, unstabilized THF (1 mL) under N_2. This was stirred for 15 min at -78 °C and then allowed to warm to rt over 1 hour before being quenched with sat. aq. NH_4Cl (10 mL) and water (10 mL). The organic components were extracted with ethyl acetate (3 x 20 mL) and the combined organic layers washed with sat. brine (10 mL), dried over anhydrous Na_2SO_4, filtered, and concentrated *in vacuo*. Purification was achieved by reverse-phase flash chromatography using a gradient of 10–90% acetonitrile/water to afford a clear oil (100 mg, 0.21 mmol, 55%).

LC-MS *m/z* calc. for $C_{22}H_{26}Cl_2N_3O_3S$ $[M(^{35}Cl)+H]^+$ = 482.1, found = 482.0, t_R = 3.49 min (Method 1).

HRMS (TOF ESI$^+$) *m/z* calc. for $C_{22}H_{26}Cl_2N_3O_3S$ $[M+H]^+$ = 482.1066, found = 482.1083.
^{1}H NMR (400 MHz, CDCl3) δ 8.34 (s, 1H), 7.46 – 7.38 (m, 2H), 7.32 – 7.24 (m, 2H), 7.07 (s, 1H), 1.55 (s, 9H), 1.25 (s, 9H) (N.B. -O**H** not observed).
^{13}C NMR (101 MHz, $CDCl_3$) δ 166.29, 163.7, 156.52, 150.26, 148.68, 144.14, 139.05, 132.61, 128.78, 128.34, 119.79, 82.94, 80.53, 74.68, 28.49, 28.07.

5-((4-chlorophenyl)(2-chlorothiazol-5-yl)methyl)pyrimidine-2,4(1*H*,3*H*)-dione (22).

Following general procedure 2, **21** (117 mg, 0.38 mmol) was converted to **22** which was isolated as a white solid (22 mg, 0.06 mmol, 16%).

LC-MS (ESI+) *m/z* calc. for $C_{14}H_{10}Cl_2N_3O_2S$ $[M(^{35}Cl)+H]^+$ = 354.0, found = 354.0, t_R = 2.78 min (Method 1).
HRMS (TOF ESI+) *m/z* calc. for $C_{14}H_{10}Cl_2N_3O_2S$ $[M+H]^+$ = 353.9865, found = 353.9858.
^{1}H NMR (400 MHz, DMSO-d_6) δ 11.26 (s, 1H), 10.92 (s, 1H), 7.43 (s, 1H), 7.39 (d, J = 8.3 Hz, 2H), 7.27 (d, J = 8.2 Hz, 2H), 7.20 (s, 1H), 5.55 (s, 1H).
^{13}C NMR (101 MHz, DMSO-d_6) δ 163.68, 151.49, 150.23, 143.10, 141.03, 140.52, 140.30, 132.14, 130.21, 128.92, 113.34, 41.03.

5-((4-chlorophenyl)(2-chlorothiazol-4-yl)methyl)pyrimidine-2,4(1*H*,3*H*)-dione (23).

To a stirred solution of **22** (1 equiv., 320 mg, 0.90 mmol) in anhydrous DCE (10 mL) was added BSTFA (4 equiv., 1 mL, 3.61 mmol) under N_2. This was refluxed overnight and then cooled before the addition of iodomethane (20 equiv., 1.1 mL, 18.00 mmol). The RM was then heated to 50 °C for 18 hours. The organic components were extracted DCM (3 x 10 mL) and the combined organic layers washed with sat. brine (10 mL), dried over anhydrous Na_2SO_4, filtered, and concentrated *in vacuo*. Purification was achieved by normal-phase flash chromatography using a gradient of 10–80% ethyl acetate/cyclohexane to afford **23** as a yellow solid (208 mg, 0.56 mmol, 63%).

LC-MS (ESI+) *m/z* calc. for $C_{15}H_{12}Cl_2N_3O_2S$ $[M(^{35}Cl)+H]^+$ = 368.0, found = 368.0, t_R = 2.80 min (Method 1).
HRMS (TOF ESI+) *m/z* calc. for $C_{15}H_{12}Cl_2N_3O_2S$ $[M+H]^+$ = 368.0022, found = 368.0018; *m/z* calc. for $C_{15}H_{11}Cl_2N_3NaO_2S$ $[M+Na]^+$ = 389.9841, found = 389.9839.
^{1}H NMR (400 MHz, DMSO-d_6) δ 11.45 (s, 1H), 7.50 (s, 1H), 7.46 – 7.35 (m, 3H), 7.33 – 7.25 (m, 2H), 5.55 (s, 1H), 3.24 (s, 3H).
^{13}C NMR (101 MHz, DMSO-d_6) δ 163.30, 151.33, 150.32, 145.47, 143.08, 140.60, 140.30, 132.19, 130.23, 128.94, 113.62, 40.94, 35.93.

***bis*(4-Chlorophenyl)(2-chlorothiazol-4-yl)methanol (24).**

Following general procedure 1, ethyl 2-chlorothiazole-4-carboxylate (2.50 g, 13.05 mmol) was converted to **8**, which was isolated as a yellow oil (3.07 g, 8.28 mmol, 63%).

LC-MS (ESI+) *m/z* calc. for $C_{16}H_{11}Cl_3NOS$ $[M(^{35}Cl)+H]^+$ = 370.0, found = not observed, t_R = 3.50 min (Method 1).
HRMS (TOF ESI+) *m/z* calc. $C_{16}H_{10}Cl_3NNaOS$ $[M+Na]^+$ = 391.9441, found = 391.9435.
^{1}H NMR (400 MHz, $CDCl_3$) δ 7.35 – 7.30 (m, 4H), 7.27 – 7.23 (m, 4H), 6.66 (s, 1H) 3.71 (s, 1H).
^{13}C NMR (101 MHz, $CDCl_3$) δ 159.48, 152.58, 142.78, 133.98, 128.90, 128.37, 118.83, 78.87.

4-(*bis*(4-Chlorophenyl)methyl)-2-chlorothiazole (25).

Following general procedure 2, **24** (3.00 g, 8.09 mmol) was converted to **25** which was isolated as an orange oil (2.42 g, 6.82 mmol, 84%).

LC-MS (ESI+) *m/z* calc. for $C_{16}H_{11}Cl_3NS$ [M(2(^{35}Cl)+^{37}Cl)+H]$^+$ = 356.0, found = 356.0, t_R = 3.55 min (Method 1).
HRMS (TOF ESI+) *m/z* calc. $C_{16}H_{11}Cl_3NS$ [M+H]$^+$ = 353.9672, found = 353.9666.
^{1}H NMR (400 MHz, $CDCl_3$) δ 7.38 – 7.26 (m, 4H), 7.15 – 7.06 (m, 4H), 6.65 (d, *J* = 1.0 Hz, 1H), 5.53 (s, 1H).
^{13}C NMR (101 MHz, $CDCl_3$) δ 156.86, 152.13, 139.80, 133.04, 130.24, 128.85, 118.55, 77.35, 77.03, 76.71, 52.49.

***bis*(4-Chlorophenyl)(2-chlorothiazol-5-yl)methanol (26).**

Following general procedure 1, ethyl 2-chlorothiazole-5-carboxylate (3.50 g, 18.26 mmol) was converted to **26** which was isolated as a yellow solid (3.94 g, 10.63 mmol, 58%).

LC-MS (ESI+) *m/z* calc. $C_{16}H_{11}Cl_3NOS$ [M(^{35}Cl)+H]$^+$ = 370.0, found = 369.8, t_R = 3.69 min (Method 1).
HRMS (TOF ESI+) *m/z* calc. $C_{16}H_{11}Cl_3NOS$ [M+H]$^+$ = 369.9621, found = 369.9623.
^{1}H NMR (400 MHz, $CDCl_3$) δ 7.39 – 7.32 (m, 4H), 7.33 – 7.25 (m, 4H), 7.07 (s, 1H) (N.B. -O**H** not observed).
^{13}C NMR (101 MHz, $CDCl_3$) δ: 153.07, 148.37, 143.05, 139.81, 134.57, 128.71, 128.38, 78.10, 77.36, 77.04, 76.72.

5-(*bis*(4-Chlorophenyl)methyl)-2-chlorothiazole (27).

Following general procedure 2, **26** (4.09 g, 11.03 mmol) was converted to **27** which was isolated as a red oil (3.21 g, 9.05 mmol, 82%).

LC-MS (ESI+) *m/z* calc. for $C_{16}H_{11}Cl_3NS$ [M(2(^{35}Cl)+^{37}Cl)+H]$^+$ = 356.0, found = 356.1, t_R = 3.91 min (Method 1).
HRMS (TOF ESI+) *m/z* calc. $C_{16}H_{11}Cl_3NS$ [M+H]$^+$ = 353.9672, found = 353.9679.
^{1}H NMR (400 MHz, $CDCl_3$) δ 7.37 – 7.29 (m, 4H), 7.18 – 7.11 (m, 4H), 7.10 (d, *J* = 1.2 Hz, 1H), 5.55 (s, 1H).
^{13}C NMR (101 MHz, $CDCl_3$) δ 151.77, 143.74, 140.02, 139.96, 133.58, 129.84, 129.11, 77.41, 77.10, 76.78, 48.69.

4-((5-(bis(4-chlorophenyl)methyl)thiazol-2-yl)amino)butanoic acid (28).

Following general procedure 3, **27** (115 mg, 0.32 mmol) was converted to **28**. Purification was achieved by reverse-phase flash chromatography using a gradient of 10–90% acetonitrile/water to afford a yellow oil (75 mg, 0.18 mmol, 56%).

LC-MS *m/z* calc. for $C_{20}H_{19}Cl_2N_2O_2S$ [M(^{35}Cl)+H]$^+$ = 421.1, found = 420.8, t_R = 2.80 min (Method 1).
HRMS (TOF ESI$^+$) *m/z* calc. $C_{20}H_{19}Cl_2N_2O_2S$ [M+H]$^+$ = 421.0539, found = 421.0535; *m/z* calc. for $C_{20}H_{18}Cl_2N_2NaO_2S$ [M+Na]$^+$ = 443.0358, found = 443.0337.

^{1}H NMR (400 MHz, $CDCl_3$) δ 7.35 (d, *J* = 8.1 Hz, 4H), 7.14 (d, *J* = 8.1 Hz, 4H), 6.60 (s, 1H), 5.35 (s, 1H), 3.36 (t, *J* = 6.9 Hz, 2H), 2.46 (t, *J* = 6.7 Hz, 2H), 2.06 – 1.98 (m, 2H) (N.B. COO**H** and N**H** not observed).
^{13}C NMR (101 MHz, DMSO) δ 171.31, 165.17, 133.79, 129.27, 125.04, 124.59, 122.44, 121.62, 43.68, 42.47, 26.38, 18.55.

2-(2-((5-(bis(4-chlorophenyl)methyl)thiazol-2-yl)amino)ethoxy)acetic acid (29).

Following general procedure 3, **27** (250 mg, 0.70 mmol) was converted to **29**. Purification was achieved by reverse-phase flash chromatography using a gradient of 10–90% acetonitrile/water followed by normal-phase flash chromatography of 0–10% MeOH/DCM to afford a yellow oil (12 mg, 0.03 mmol, 4%).

LC-MS *m/z* calc. for $C_{20}H_{19}Cl_2N_2O_3S$ $[M(^{35}Cl)+H]^+$ = 437.0, found = 436.8, t_R = 2.80 min (Method 1).
HRMS (TOF ESI^+) *m/z* calc. for $C_{20}H_{19}Cl_2N_2O_3S$ $[M+H]^+$ = 437.0488, found = 437.0480.
^{1}H NMR (400 MHz, DMSO) δ 7.80 (s, 1H), 7.43 – 7.35 (m, 4H), 7.29 – 7.18 (m, 4H), 6.52 (d, *J* = 1.0 Hz, 1H), 5.62 (s, 1H), 3.87 (s, 2H), 3.60 (t, *J* = 5.6 Hz, 2H), 3.33 (t, *J* = 5.6 Hz, 2H) (N.B. -COO**H** not observed).
^{13}C NMR (101 MHz, DMSO) δ 173.42, 169.14, 142.46, 137.40, 131.86, 130.61, 128.96, 127.09, 69.17, 47.67, 44.25. (N.B. one peak missing).

5-((5-(bis(4-chlorophenyl)methyl)thiazol-2-yl)amino)pentanoic acid (30).

Following general procedure 3, **29** (119 mg, 0.34 mmol) was converted to **30** and purified by reverse-phase flash chromatography using a gradient of 10–90% acetonitrile/water followed by lyophilisation to afford a yellow oil (18 mg, 0.04 mmol, 12%).

LC-MS *m/z* calc. for $C_{21}H_{21}Cl_2N_2O_2S$ $[M(^{35}Cl)+H]^+$ = 435.06, found = 435.7, t_R = 2.90 min (Method 1).
HRMS (TOF ESI^+) *m/z* calc. $C_{21}H_{21}Cl_2N_2O_2S$ $[M+H]^+$ = 435.0695, found = 435.0686; *m/z* calc. for $C_{21}H_{20}Cl_2N_2NaO_2S$ $[M+Na]^+$ = 457.0515, found = 457.0509.
^{1}H NMR (400 MHz, $CDCl_3$) δ 7.33 – 7.28 (m, 4H), 7.18 – 7.10 (m, 4H), 6.47 (s, 1H), 5.37 (s, 1H), 4.73 (s, 1H), 3.13 (t, *J* = 5.7 Hz, 2H), 2.36 (t, *J* = 6.4 Hz, 2H), 1.85 – 1.66 (m, 4H) (N.B. -COO**H** not observed).
^{13}C NMR (101 MHz, $CDCl_3$) δ 178.47, 172.62, 140.55, 134.83, 133.11, 129.93, 128.83, 126.45, 48.59, 46.45, 33.89, 28.40, 22.34.

Ethyl 5-((5-(bis(4-chlorophenyl)methyl)thiazol-2-yl)amino)pentanoate (31).

Following general procedure 3, **27** (89 mg, 0.25 mmol) was converted to **31**. Purification was achieved by reverse-phase flash chromatography using a gradient of 10–90% acetonitrile/water followed by normal-phase flash chromatography of 0–10% MeOH/DCM to afford a yellow oil (8 mg, 0.02 mmol, 7%).

LC-MS *m/z* calc. for $C_{23}H_{25}Cl_2N_2O_2S$ $[M(^{35}Cl)+H]^+$ = 463.1, found = 462.7, t_R = 3.04 min (Method 1).
HRMS (TOF ESI^+) *m/z* calc. for $C_{23}H_{25}Cl_2N_2O_2S$ $[M+H]^+$ = 463.1008, found = 463.1004.
^{1}H NMR (400 MHz, DMSO) δ 7.49 (t, *J* = 5.5 Hz, 1H), 7.43 – 7.35 (m, 4H), 7.27 – 7.19 (m, 4H), 6.51 (d, *J* = 1.2 Hz, 1H), 5.61 (s, 1H), 4.04 (q, *J* = 7.1 Hz, 2H), 3.14 (q, *J* = 6.2 Hz, 2H), 2.29 (t, *J* = 6.9 Hz, 2H), 1.53 (ddt, *J* = 12.4, 9.4, 5.0 Hz, 4H), 1.16 (t, *J* = 7.1 Hz, 3H).
^{13}C NMR (101 MHz, DMSO) δ 173.24, 169.28, 142.48, 137.52, 131.86, 130.61, 128.96, 126.85, 60.15, 47.69, 44.20, 33.63, 28.54, 22.45, 14.59.

6-((5-(bis(4-chlorophenyl)methyl)thiazol-2-yl)amino)hexanoic acid (32).

Following general procedure 3, **27** (250 mg, 0.70 mmol) was converted to **32** and purified by reverse-phase flash chromatography using a gradient of 10–90% acetonitrile/water to afford a white solid (36 mg, 0.08 mmol, 18%).

LC-MS *m/z* calc. for $C_{22}H_{23}Cl_2N_2O_2S$ $[M(^{35}Cl)+H]^+$ = 449.1, found = 448.7, t_R = 2.83 min (Method 1).
HRMS (TOF ESI$^+$) *m/z* calc. for $C_{22}H_{23}Cl_2N_2O_2S$ $[M+H]^+$ = 449.0852, found = 449.0862; *m/z* calc. for $C_{22}H_{22}Cl_2N_2NaO_2S$ $[M+Na]^+$ = 471.0671, found = 471.0666.
^{1}H NMR (400 MHz, $CDCl_3$) δ 7.34 – 7.27 (m, 4H), 7.18 – 7.09 (m, 4H), 6.46 (d, *J* = 1.3 Hz, 1H), 5.36 (s, 1H), 3.13 (t, *J* = 6.4 Hz, 2H), 2.65 (s, 1H), 2.32 (t, *J* = 6.9 Hz, 2H), 1.73 – 1.62 (m, 4H), 1.54 – 1.40 (m, 2H) (N.B. -COO**H** not observed).
^{13}C NMR (101 MHz, $CDCl_3$) δ 178.79, 172.31, 140.54, 134.78, 133.10, 129.93, 128.84, 126.40, 48.58, 46.65, 34.76, 28.66, 26.55, 24.71.

7-((5-(bis(4-chlorophenyl)methyl)thiazol-2-yl)amino)heptanoic acid (33).

Following general procedure 3, **27** (250 mg, 0.70 mmol) was converted to **33** and purified by reverse-phase flash chromatography using a gradient of 10–90% acetonitrile/water to afford a white solid (21 mg, 0.05 mmol, 11%).

LC-MS *m/z* calc. for $C_{23}H_{25}Cl_2N_2O_2S$ $[M(^{35}Cl)+H]^+$ = 463.1, found = 462.7, t_R = 2.85 min (Method 1).
HRMS (TOF ESI$^+$) *m/z* calc. for $C_{23}H_{25}Cl_2N_2O_2S$ $[M+H]^+$ = 463.1008, found = 463.1016; *m/z* calc. for $C_{23}H_{24}Cl_2N_2NaO_2S$ $[M+Na]^+$ = 485.0828, found = 485.0844.
^{1}H NMR (400 MHz, $CDCl_3$) δ 7.34 – 7.26 (m, 4H), 7.18 – 7.09 (m, 4H), 6.47 (d, *J* = 1.3 Hz, 1H), 5.36 (s, 1H), 3.10 (t, *J* = 6.3 Hz, 2H), 2.36 – 2.26 (m, 2H), 1.66 (h, *J* = 6.9 Hz, 4H), 1.50 – 1.34 (m, 4H) (N.B. -COO**H** and -N**H** not observed).
^{13}C NMR (101 MHz, $CDCl_3$) δ 179.01, 172.46, 140.60, 135.02, 133.08, 129.93, 128.82, 126.43, 48.60, 46.55, 34.67, 28.78, 28.59, 26.62, 24.89.

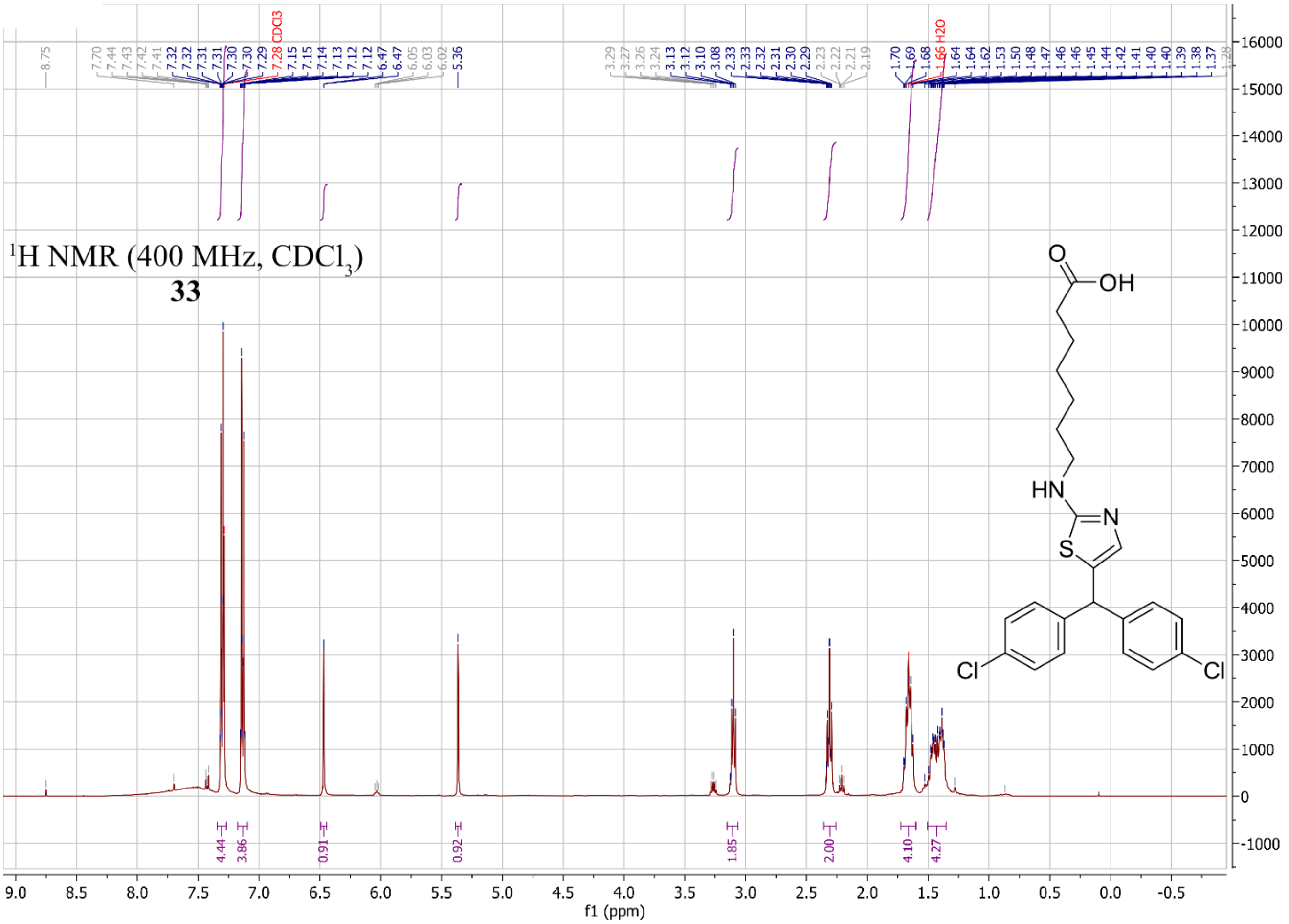

7-((5-(bis(4-chlorophenyl)methyl)thiazol-2-yl)amino)-*N*-(1*H*-tetrazol-5-yl)heptanamide (34).

To a stirred solution of **33** (1 equiv., 40 mg, 0.09 mmol) in DMF (4 mL) was added DIPEA (10 equiv., 150 µL, 0.86 mmol), 5-aminotetrazole monohydrate (6 equiv., 53 mg, 0.52 mmol), and PyBroP (3 equiv., 120 mg, 0.26 mmol). The RM was stirred overnight and then concentrated *in vacuo* to dryness. Purification was achieved by normal-phase flash chromatography using a gradient of 0–15% MeOH/DCM followed by reverse-phase flash chromatography 10–90% acetonitrile/water to afford **34** as a white solid (4 mg, 7.54 µmol, 9%).

LC-MS *m/z* calc. for $C_{24}H_{26}Cl_2N_7OS$ $[M(^{35}Cl)+H]^+$ = 530.1, found = 529.7, t_R = 2.81 min (Method 1).
HRMS (TOF ESI$^+$) *m/z* calc. for $C_{24}H_{26}Cl_2N_7OS$ $[M+H]^+$ = 530.1291, found = 530.1312.
^{1}H NMR (400 MHz, DMSO) δ 11.81 (s, 1H), 7.47 (t, *J* = 5.4 Hz, 1H), 7.43 – 7.34 (m, 4H), 7.27 – 7.18 (m, 4H), 6.51 (s, 1H), 5.61 (s, 1H), 3.13 (q, *J* = 6.4 Hz, 2H), 2.42 (t, *J* = 7.3 Hz, 2H), 1.59 (m, 2H), 1.50 (m, 2H), 1.35 – 1.28 (m, 4H) (N.B. tetrazole-N**H** not observed).
^{13}C NMR (101 MHz, DMSO) δ 172.42, 169.34, 150.31, 142.48, 137.52, 131.85, 130.61, 128.96, 126.77, 47.69, 44.60, 35.40, 28.99, 28.65, 26.62, 24.88.

***tert*-butyl (*S*)-5-((5-(bis(4-chlorophenyl)methyl)thiazol-2-yl)amino)-2-((*tert*-butoxycarbonyl)amino)pentanoate (35).**

Following general procedure 3, **27** (112 mg, 0.32 mmol) was converted to **35** and purified by reverse-phase flash chromatography using a gradient of 10–90% acetonitrile/water to afford a white solid (117 mg, 0.19 mmol, 61%).

LC-MS *m/z* calc. for $C_{30}H_{38}Cl_2N_3O_4S$ $[M+H]^+$ = 606.2, found = 605.9, t_R = 3.19 min (Method 1).
HRMS (TOF ESI$^+$) *m/z* calc. $C_{30}H_{38}Cl_2N_3O_4S$ $[M+H]^+$ = 606.1955, found = 606.1956; *m/z* calc. $C_{30}H_{37}Cl_2N_3NaO_4S$ $[M+Na]^+$ = 628.1774, found = 628.1757.
^{1}H NMR (400 MHz, DMSO) δ 7.48 (t, *J* = 5.4 Hz, 1H), 7.41 – 7.37 (m, 4H), 7.25 – 7.20 (m, 4H), 7.11 (d, *J* = 7.7 Hz, 1H), 6.51 (d, *J* = 1.0 Hz, 1H), 5.62 (s, 1H), 3.79 – 3.74 (m, 1H), 3.12 (q, *J* = 6.2 Hz, 2H), 1.66 (d, *J* = 9.4 Hz, 1H), 1.60 – 1.52 (m, 3H), 1.37 (d, *J* = 3.4 Hz, 18H).
^{13}C NMR (101 MHz, DMSO) δ 172.18, 169.28, 155.98, 142.47, 137.52, 131.86, 130.60, 128.95, 126.88, 80.66, 78.49, 54.57, 47.68, 44.19, 28.66, 28.09, 25.75.

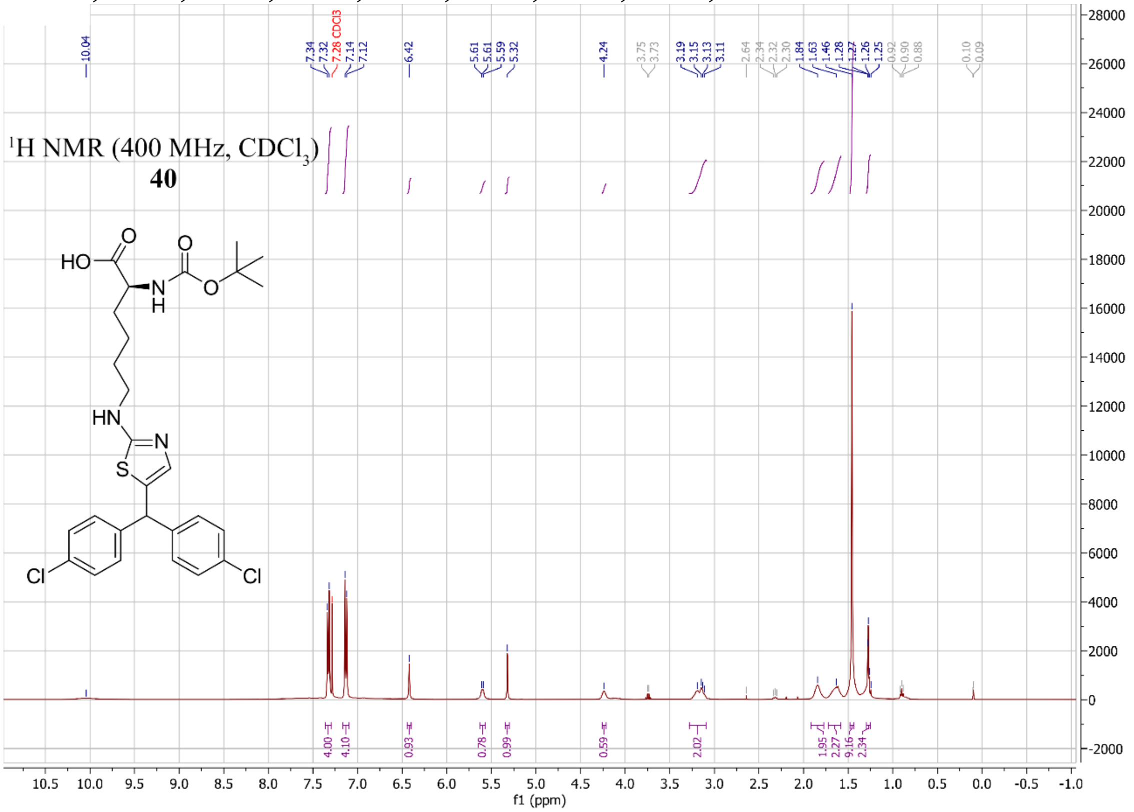

(*S*,*E*)-5-((5-(bis(4-chlorophenyl)methyl)thiazol-2-yl)amino)-2-(6-(2-(4-(2-(5,5-difluoro-7-(thiophen-2-yl)-5*H*-4λ^4,5λ^4-dipyrrolo[1,2-*c*:2',1'-*f*][1,3,2]diazaborinin-3-yl)vinyl)phenoxy)acetamido)hexanamido)pentanoic acid (37).

To a stirred solution of **35** (1 equiv., 1 mg, 1.65 µmol) in DCM (0.4 mL) was added TFA (0.4 mL) and then stirred at rt for 1h. The RM was diluted with toluene (15 mL) and concentrated *in vacuo* to one-fifth of the volume, this was repeated three times before it was concentrated to dryness. Then, following general procedure 4, the deprotected amine was converted to the BODIPY630/650–X conjugate **37**. This was purified by semi-preparative RP HPLC (Method E) with a retention time of 7.5 min and freeze-dried to a blue solid (0.78 mg, 0.783 µmol, 47%).

LC-MS *m/z* calc. for $C_{50}H_{48}BCl_2F_2N_6O_5S_2$ $[M+H]^+$ = 995.3, found = 995.1, t_R = 6.99 min (Method 2).

HRMS (TOF ESI+) *m/z* calc. $C_{50}H_{47}BCl_2F_2N_6O_5S_2$ $[M-H]^-$ = 994.2493, found = 994.2553.
Analytical RP-HPLC (Method F) t_R = 18.6 min, purity 99%.

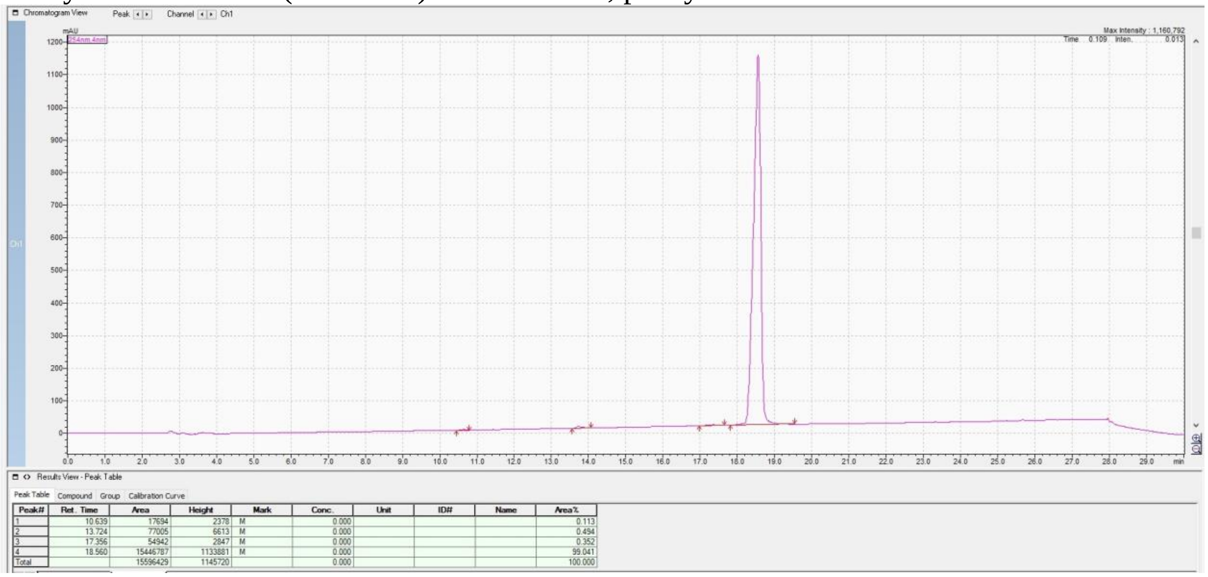

(*S*)-5-((5-(bis(4-chlorophenyl)methyl)thiazol-2-yl)amino)-2-(6-(3-(5,5-difluoro-7,9-dimethyl-5H-4λ^4,5λ^4-dipyrrolo[1,2-*c*:2',1'-*f*][1,3,2]diazaborinin-3-yl)propanamido)hexanamido)pentanoic acid (38).

To a stirred solution of **35** (1 equiv., 1 mg, 1.65 µmol) in DCM (0.4 mL) was added TFA (0.4 mL) and then stirred at rt for 1h. The RM was diluted with toluene (15 mL) and concentrated *in vacuo* to one-fifth of the volume, this was repeated three times before it was concentrated to dryness. Then, following general procedure 4, the deprotected amine was converted to the BODIPYFL-X conjugate **38**. This was purified by analytical RP HPLC (Method B) with a retention time of 8.10 min and freeze-dried to a red solid (0.97 mg, 1.16 µmol, 70%).

LC-MS *m/z* calc. for $C_{41}H_{46}BCl_2F_2N_6O_4S$ $[M+H]^+$ = 837.3, found = 837.3, t_R = 6.21 min (Method 2).
HRMS (TOF ESI+) *m/z* calc. $C_{41}H_{46}BCl_2F_2N_6O_4S$ $[M+H]^+$ = 837.2734, found = 837.2716; *m/z* calc. for $C_{41}H_{45}BCl_2F_2N_6NaO_4S$ $[M+Na]^+$ = 859.2553, found = 859.2569.
Analytical RP-HPLC (Method F) t_R = 15.7 min, purity 97%.

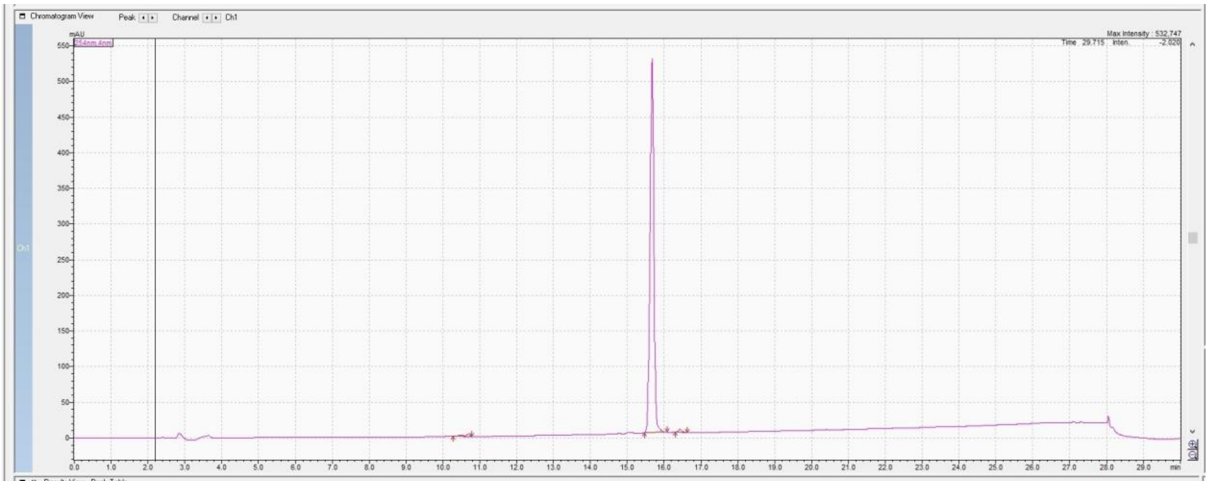

Results View - Peak Table

Peak Table | Compound | Group | Calibration Curve

Peak#	Ret. Time	Area	Height	Mark	Conc.	Unit	ID#	Name	Area%
1	10.678	32041	3549	M	0.000				0.839
2	15.678	3760656	525310	M	0.000				98.493
3	16.433	25497	3970	M	0.000				0.668
Total		3818194	532829		0.000				100.000

(*S*)-5-((4-((5-(bis(4-chlorophenyl)methyl)thiazol-2-yl)amino)-1-carboxybutyl)carbamoyl)-2-(6-(dimethylamino)-3-(dimethyliminio)-3*H*-xanthen-9-yl)benzoate (39).

To a stirred solution of **35** (1 equiv., 1 mg, 1.65 µmol) in DCM (0.4 mL) was added TFA (0.4 mL) and then stirred at rt for 1h. The RM was diluted with toluene (15 mL) and concentrated *in vacuo* to one-fifth of the volume, this was repeated three times before it was concentrated to dryness. Then, following general procedure 4, the deprotected amine was converted to the 5-TAMRA conjugate **39**. This was purified by semi-preparative RP HPLC (Method D) with a retention time of 8.2 min and freeze-dried to a purple solid (1.21 mg, 1.40 µmol, 85%).

LC-MS *m/z* calc. for $C_{46}H_{42}Cl_2N_5O_6S$ $[M+H]^+$ = 862.22, found = 861.9, t_R = 5.71 min (Method 2).
HRMS (TOF ESI$^+$) *m/z* calc. $C_{46}H_{42}Cl_2N_5O_6S$ $[M+H]^+$ = 862.2227, found = 862.2236.
Analytical RP-HPLC (Method F) t_R = 12.3 min, purity 95%.

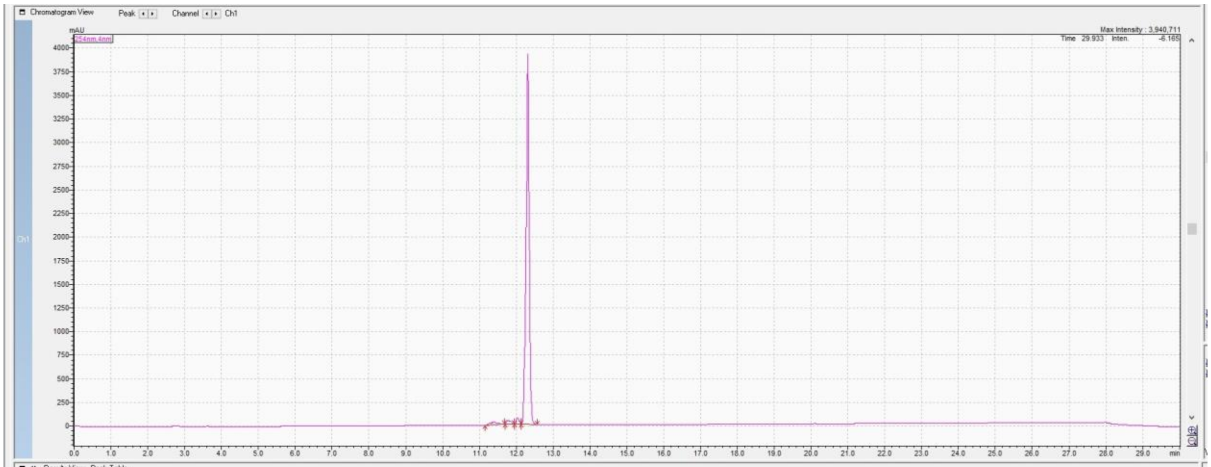

Results View - Peak Table

Peak Table | Compound | Group | Calibration Curve

Peak#	Ret. Time	Area	Height	Mark	Conc.	Unit	ID#	Name	Area%
1	11.391	388259	31685	M	0.000				1.587
2	11.771	333812	41245	M	0.000				1.365
3	12.039	337153	65461	M	0.000				1.378
4	12.314	23401369	3920481	M	0.000				95.670
Total		24460592	4058873		0.000				100.000

N^6-(5-(bis(4-chlorophenyl)methyl)thiazol-2-yl)-N^2-(*tert*-butoxycarbonyl)-*L*-lysine (40).

Following general procedure 3, **27** (112 mg, 0.32 mmol) was converted to **40** and purified by reverse-phase flash chromatography using a gradient of 10–90% acetonitrile/water followed by normal-phase flash chromatography of 0–10% MeOH/DCM to afford a yellow oil (32 mg, 0.06 mmol, 21%).

LC-MS *m/z* calc. for $C_{27}H_{32}Cl_2N_3O_4S$ $[M(^{35}Cl+^{37}Cl)+H]^+$ = 566.1, found = 565.9, t_R = 2.93 min (Method 1).
HRMS (TOF ESI$^+$) *m/z* calc. for $C_{27}H_{32}Cl_2N_3O_4S$ $[M+H]^+$ = 564.1485 , found = 564.1493; *m/z* calc. for $C_{27}H_{31}Cl_2N_3NaO_4S$ $[M+Na]^+$ = 586.1305, found = 586.1297.
^{1}H NMR (400 MHz, $CDCl_3$) δ 10.04 (s, 1H), 7.33 (d, *J* = 8.2 Hz, 4H), 7.13 (d, *J* = 8.2 Hz, 4H), 6.42 (s, 1H), 5.63 – 5.57 (m, 1H), 5.32 (s, 1H), 4.24 (s, 1H), 3.29 – 3.08 (m, 2H), 1.84 (br. s, 2H), 1.62 (br. s, *J* = 6.4 Hz, 2H), 1.46 (s, 9H), 1.29 – 1.24 (m, 2H) (N.B. -N**H** not observed).
^{13}C NMR (101 MHz, $CDCl_3$) δ 171.82, 155.31, 139.72, 133.47, 129.86, 129.02, 126.02, 79.21, 54.30, 48.53, 46.98, 29.37, 28.45, 28.15, 21.81.

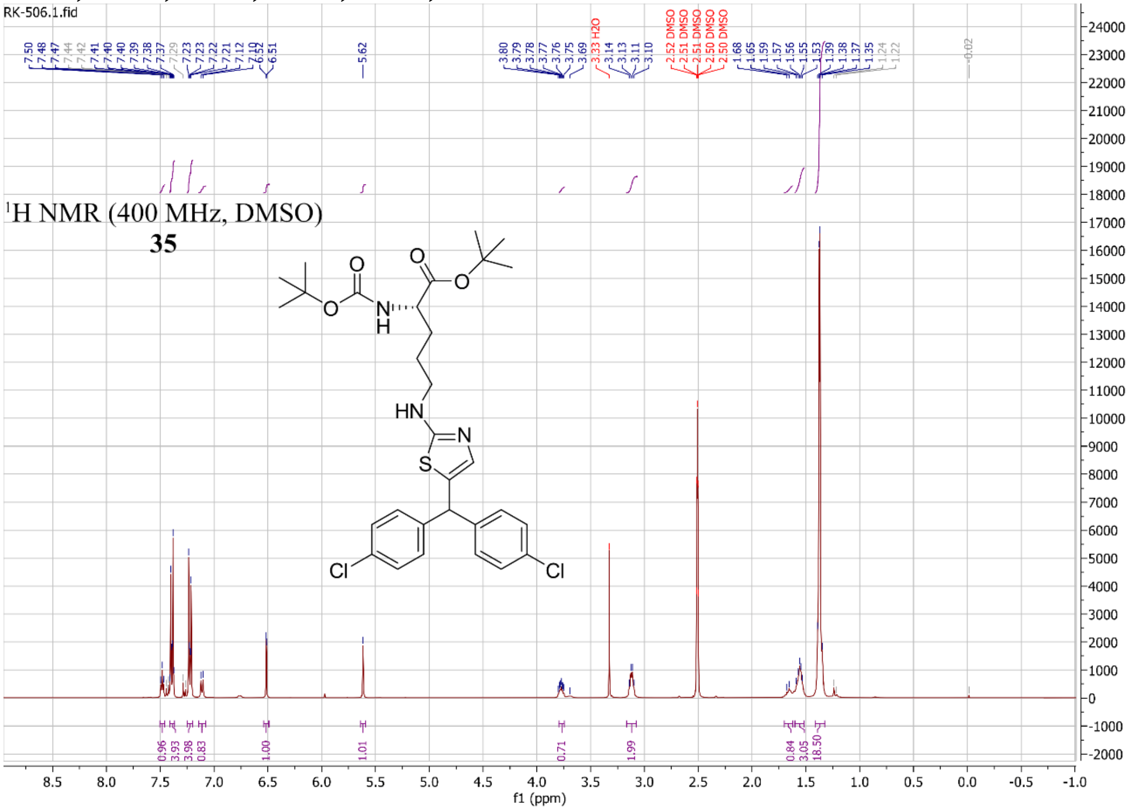

(*E*)-N^6-(5-(bis(4-chlorophenyl)methyl)thiazol-2-yl)-N^2-(6-(2-(4-(2-(5,5-difluoro-7-(thiophen-2-yl)-5*H*-4λ^4,5λ^4-dipyrrolo[1,2-*c*:2',1'-*f*][1,3,2]diazaborinin-3-yl)vinyl)phenoxy)acetamido)hexanoyl)-*L*-lysine (42).

To a stirred solution of **40** (1 equiv., 1 mg, 1.77 µmol) in DCM (0.5 mL) was added TFA (0.25 mL) and then stirred at rt for 30 min. The RM was diluted with toluene (15 mL) and concentrated *in vacuo* to one-fifth of the volume, this was repeated three times before it was concentrated to dryness. Then, following general procedure 4, the deprotected amine was converted to the BODIPY630/650–X conjugate

42. This was purified by analytical RP HPLC (Method E) with a retention time of 7.5 min and freeze-dried to a blue solid (1.75 mg, 1.73 µmol, 97%).

LC-MS *m/z* calc. for $C_{51}H_{50}BCl_2F_2N_6O_5S_2$ $[M+H]^+$ = 1009.3, found = 1009.1, t_R = 7.0 min (Method 2).
HRMS (TOF ESI$^-$) *m/z* calc. for $C_{51}H_{48}BCl_2F_2N_6O_5S_2$ $[M-H]^-$ = 1007.2650, found = 1007.2644.
Analytical RP-HPLC (Method F) t_R = 18.5 min, purity 99%.

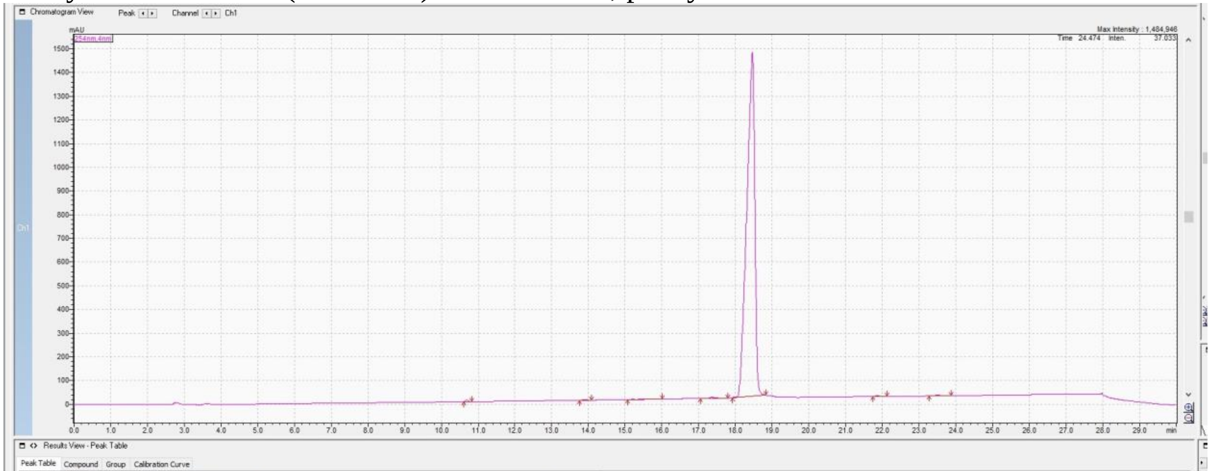

Peak#	Ret. Time	Area	Height	Mark	Conc.	Unit	ID#	Name	Area%
1	10.688	36324	7200	M	0.000				0.163
2	13.872	19877	2762	M	0.000				0.089
3	15.658	36273	1898	M	0.000				0.163
4	17.356	65719	5065	M	0.000				0.295
5	18.463	22024951	1451407	M	0.000				99.007
6	21.863	33285	3182	M	0.000				0.150
7	23.501	29400	3474	M	0.000				0.132
Total		22245829	1474988		0.000				100.000

***N*6-(5-(bis(4-chlorophenyl)methyl)thiazol-2-yl)-*N*^{2}-(6-(3-(5,5-difluoro-7,9-dimethyl-5H-4λ^4,5λ^4-dipyrrolo[1,2-*c*:2',1'-*f*][1,3,2]diazaborinin-3-yl)propanamido)hexanoyl)-*L*-lysine (43).**

To a stirred solution of **40** (1 equiv., 1 mg, 1.77 µmol) in DCM (0.5 mL) was added TFA (0.25 mL) and then stirred at rt for 30 min. The RM was diluted with toluene (15 mL) and concentrated *in vacuo* to one-fifth of the volume, this was repeated three times before it was concentrated to dryness. Then, following general procedure 4, the deprotected amine was converted to the BODIPYFL-X conjugate **43**. This was purified by analytical RP HPLC (Method B) with a retention time of 7.5 min and freeze-dried to a red solid (1.33 mg, 1.56 µmol, 88%).

LC-MS *m/z* calc. for $C_{42}H_{48}BCl_2F_2N_6O_4S$ $[M+H]^+$ = 851.6, found = 851.4, t_R = 6.24 min (Method 2).
HRMS (TOF ESI$^+$) *m/z* calc. for $C_{42}H_{48}BCl_2F_2N_6O_4S$ $[M+H]^+$ = 851.2890, found = 851.2907.
HRMS (TOF ESI$^-$) *m/z* calc. for $C_{42}H_{46}BCl_2F_2N_6O_4S$ $[M-H]^-$ = 849.2745, found = 849.2748.
Analytical RP-HPLC (Method F) t_R = 17.8 min, purity 97%.

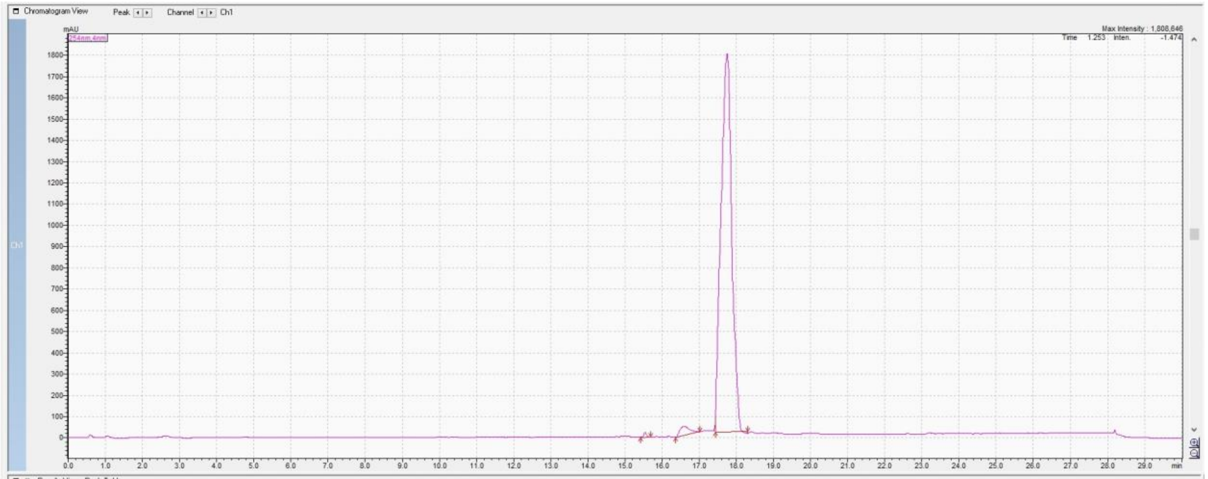

Results View - Peak Table

Peak#	Ret. Time	Area	Height	Mark	Conc.	Unit	ID#	Name	Area%
1	15.543	123835	23852	M	0.000				0.337
2	16.621	781571	41287	M	0.000				2.125
3	17.753	35871652	1780550	M	0.000				97.538
Total		36777058	1845689		0.000				100.000

((*S*)-5-((5-((5-(bis(4-chlorophenyl)methyl)thiazol-2-yl)amino)-1-carboxypentyl)carbamoyl)-2-(6-(dimethylamino)-3-(dimethyliminio)-3*H*-xanthen-9-yl)benzoate (44).

To a stirred solution of **40** (1 equiv., 1.2 mg, 2.58 µmol) in DCM (0.5 mL) was added TFA (0.25 mL) and then stirred at rt for 30 min. The RM was diluted with toluene (15 mL) and concentrated *in vacuo* to one-fifth of the volume, this was repeated three times before it was concentrated to dryness. Then, following general procedure 4, the deprotected amine was converted the 5-TAMRA conjugate **44**. This was purified by analytical RP HPLC (Method D) with a retention time of 8.1 min and freeze-dried to a purple solid (1.50 mg, 1.71 µmol, 66%).

LC-MS *m/z* calc. for $C_{47}H_{44}Cl_2N_5O_6S$ $[M+H]^+$ = 876.2, found = 876.0, t_R = 5.73 min (Method 2).
HRMS (TOF ESI$^+$) *m/z* calc. for $C_{47}H_{44}Cl_2N_5O_6S$ $[M+H]^+$ = 876.2384, found = 876.2403.
Analytical RP-HPLC (Method F) t_R = 12.2 min, purity 98%.

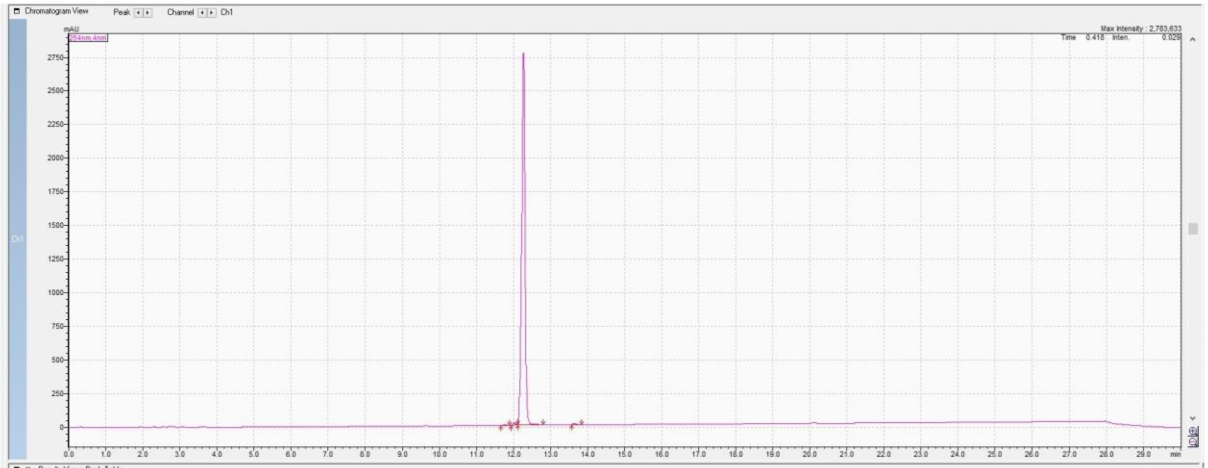

Results View - Peak Table

Peak#	Ret. Time	Area	Height	Mark	Conc.	Unit	ID#	Name	Area%
1	11.768	25031	4774	M	0.000				0.145
2	12.017	76006	14754	M	0.000				0.440
3	12.272	17094258	2766224	M	0.000				98.922
4	13.640	85189	11491	M	0.000				0.493
Total		17280484	2797243		0.000				100.000

Docking

All programmes were used with the default parameters. The $P2Y_2R$ homology model was kindly supplied by Dr Müller. The $P2Y_2R$ homology model with AR-C118925 bound was prepared for docking using OpenEye Make Receptor v3.0.0. OpenEye OMEGA2 was used to convert compound SMILES to 3D conformers. Docking into the $P2Y_2R$ homology model was performed using OEDocking HYBRID with the Chemguass4 scoring function.

Pharmacology: Materials and Methods - No unexpected or unusually high safety hazards were encountered.

AR-C118925 and UTPγS were purchased from Tocris Bioscience (Bristol, UK). CXCL12-AF647 was purchased from ALMAC (Loughborough, UK). AMD3100 was purchased from Merck Millipore.

Cell Culture

Clonal 1321N1 cells which stably expressed recombinant N-terminal tagged NanoLuc-$P2Y_2$ (NLuc-$P2Y_2R$-1321N1) and 1321N1 cells which stably expressed $P2Y_2$ ($P2Y_2R$-1321N1) were maintained in Dulbecco's Modified Eagle's Medium (DMEM) - High Glucose, supplemented with 10% fetal calf serum (FCS) and 2 mM L-glutamine at 37 °C and 5% CO_2. Cells were passaged at 80-90% confluency using Phosphate Buffered Saline (PBS) and 2X Trypsin-EDTA (0.25% w/v in versene).

Membrane Preparation

NLuc-$P2Y_2R$ expressing 1321N1 cells were grown to 90-100% confluency in three T-175 cm^2 culture flasks with DMEM High Glucose/10% FCS/2 mM L-glutamine. The media was replaced with PBS and cells removed from the surface of the flask by scraping. Cells were centrifuged at 1,500 x *g* for 10 min at 4 °C and the resulting pellets were stored at -80 °C. The thawed pellets were resuspended and combined in PBS before being homogenised with an electronic handheld IKA T10 Ultra Turrax homogeniser in 12 x 2 s bursts at 22,000 rpm. This was then centrifuged 1,500 x *g* for 20 min at 4 °C to remove unbroken cells and nuclei. The supernatant was further centrifuged at 40,000 x *g* for 30 min at 4 °C to pellet the membranes. The pellet was resuspended in 2 mL of PBS and then, in a boroscillate glass homogeniser mortar, homogenised with 22 passes of a serrated pestle attached to a KA RW16 overhead stirrer at 1,000 rpm. Protein concentration was determined using the Pierce™ BCA Protein Assay kit and Dynex Technologies 4.25 plate reader. Membrane preparations were then stored at -80 °C. NLuc-$CXCR_4$ membranes were prepared using the same method from a clonal NLuc-$CXCR_4$ HEK293 cell line.

NanoBRET Assays

For membrane experiments, defrosted membranes were plated in white clear bottom 96-well microplates (655098, Grenier Bio-One; Stonehouse, UK) at 5 µg per well diluted in HBSS with different concentrations of BSA and saponin depending on the fluorescent ligand. For experiments with **3** 0.01% BSA was used, for **37** and **42** 0.1% BSA with 1 mg/mL saponin, for **38** and **43** 0.05% BSA with 1 mg/mL saponin, and for **39** and **44** 0.01% BSA with 1 mg/mL saponin. After addition of the required concentration of fluorescent ligand with or without competing antagonist, the membranes were incubated for 1 hour at 37 °C without CO_2. Following incubation, the NanoLuc substrate furimazine (Promega Corporation) was added as a final well dilution of 1:400. The luminescence and fluorescence emissions were simultaneously measured using a PHERAstar FS plate reader (BMG Labtech) at room tempera-

ture. For assays involving **3**, **38**, and **43** measurements were made at 475 nm (30 nm bandpass) and 535 nm (30 nm bandpass), with the raw BRET ratios calculated through division of the 535 nm emissions by the 475 nm emissions. For assays involving **39** and **44** measurements were made at 450 nm (80 nm bandpass) and > 550 nm (long pass), with the raw BRET ratios calculated through division of the > 550 nm emissions by the 480 nm emissions. For assays involving **37** and **42** measurements were made at 460 nm (80 nm bandpass) and > 610 nm (long pass), with the raw BRET ratios calculated through division of the > 610 nm emissions by the 460 nm emissions.

Calcium-Mobilisation Assay

$P2Y_2R$ expressing 1321N1 cells were seeded into black clear bottom 96-well microplates (655090, Grenier Bio-One; Stonehouse, UK) at a density of 20,000 cells per well (in DMEM supplemented with 10% FCS) 24 hours prior to the experiment. The media was removed from each well and replaced with HBSS containing 2.5 mM probenecid, 2.3 μM Fluo 4AM, 0.023% pluronic acid, 0.5 mM Brilliant Black, 1 U/mL apyrase, and the required concentration of ligand under investigation before incubation for 1 hour at 37 °C without CO_2. After incubation, the fluorescence was measured (excitation: 485 nm, emission: 525 nm) every 1.52 seconds for up to 200 seconds after the addition of UTPγS at 15 seconds using a FLEXstation 3 (Molecular Devices) plate reader.

Data Analysis

All the data was analysed using GraphPad Prism 9.

The total and non-specific saturation binding curves were fitted simultaneously using equation 1:

$$\text{BRET ratio} = \frac{B_{\text{max}} \times [\text{b}]}{[\text{b}] + K_{\text{d}}} + ((M \times [\text{b}]) + C)$$

where B_{max} is the maximum specific BRET signal, [b] is the nanomolar concentration of the fluorescent ligand, K_d is the equilibrium dissociation constant in nanomolar, M is the slope of the non-specific binding component, and C is the intercept with the Y-axis. Where the non-specific binding curve was linear, background and non-specific binding components were shared across all data sets with non-specific binding constrained to be greater than 0. When the non-specific binding curves showed saturable binding, total and non-specific binding curves were both fitted to the above equation with only background BRET shared between the data sets.

The specific saturation binding was calculated by deducting the non-specific binding and the binding curve fitted using equation 2:

$$\text{BRET ratio} = \frac{B_{\text{max}} \times [\text{b}]}{[\text{b}] + K_{\text{d}}}$$

where B_{max} is the maximum specific BRET signal, [b] is the nanomolar concentration of the fluorescent ligand and K_d is the equilibrium dissociation constant in nanomolar. The competition binding curve was fitted using equation 3:

$$K_i = \frac{\text{IC}_{50}}{1 + \frac{[\text{L}]}{K_{\text{d}}}}$$

where [L] is the nanomolar concentration of fluorescent ligand, and K_d is the equilibrium dissociation constant of the fluorescent ligand in nanomolar. The IC_{50} was calculated as in equation 4:

$$\% \text{ inhibition of specific binding} = \frac{100 \times [A]}{[A] + IC_{50}}$$

where [A] is the concentration of competing antagonist, and the IC_{50} is the molar concentration of competing antagonist required to inhibit 50% of the specific binding of the fluorescent ligand at a given concentration ([L]).

1. Rafehi, M.; Neumann, A.; Baqi, Y.; Malik, E. M.; Wiese, M.; Namasivayam, V.; Müller, C. E., Molecular Recognition of Agonists and Antagonists by the Nucleotide-Activated G Protein-Coupled P2Y2 Receptor. *Journal of Medicinal Chemistry* **2017,** *60* (20), 8425-8440.
